# The potential of anti-malarial compounds derived from African medicinal plants: a review of pharmacological evaluations from 2013 to 2019

**DOI:** 10.1186/s12936-020-03231-7

**Published:** 2020-05-18

**Authors:** Boris D. Bekono, Fidele Ntie-Kang, Pascal Amoa Onguéné, Lydia L. Lifongo, Wolfgang Sippl, Karin Fester, Luc C. O. Owono

**Affiliations:** 1 Department of Physics, Ecole Normale Supérieure, University of Yaoundé I, P. O. Box 47, Yaoundé, Cameroon; 2 Department of Chemistry, Faculty of Science, University of Buea, P. O. Box 63, Buea, Cameroon; 3 Department of Pharmaceutical Chemistry, Martin-Luther University of Halle-Wittenberg, Kurt-Mothes Str. 3, 06120 Halle (Saale), Germany; 4 Institut für Botanik, Technische Universität Dresden, Zellescher Weg 20b, 01062 Dresden, Germany; 5 Department of Chemistry, University Institute of Wood Technology Mbalmayo, University of Yaoundé I, BP 50, Mbalmayo, Cameroon; 6 Faculty of Natural and Environmental Sciences, Zittau/Görlitz University of Applied Sciences, Theodor-Körner-Allee 16, 02763 Zittau, Germany

**Keywords:** Africa, Malaria, Medicinal plants, Natural products, Traditional medicine

## Abstract

**Background:**

African Traditional Medicine (ATM) is used for the healthcare of about 80% of the rural populations of the continent of Africa. The practices of ATM make use of plant-products, which are known to contain plant-based secondary metabolites or natural products (NPs), likely to play key roles in drug discovery, particularly as lead compounds. For various reasons, including resistance of strains of *Plasmodium* to known anti-malarial drugs, local African populations often resort to plant-based treatments and/or a combination of this and standard anti-malarial regimens. Emphasis has been laid in this review to present the anti-malarial virtue of the most recently published phytochemicals or natural products, which have been tested by in vitro and in vivo assays.

**Methods:**

The data was based on the current version of the African Compound Libraries, which are constantly being updated based on inputs from journal articles and student theses (M.Sc/Ph.D) from African University libraries. Emphasis was laid on data published after 2012. In order to carry out the original data collection, currently being included in the African Compounds Database, individual journal websites were queried using the country names in Africa as search terms. Over 40,000 articles “hits” were originally retrieved, then reduced to about 9000 articles. The retained articles/theses was further queried with the search terms “malaria”, “malarial”, “plasmodium”, “plasmodial” and a combination of them, resulting in over 500 articles. Those including compounds with anti-malarial activities for which the measured activities fell within the established cut off values numbered 55, which were all cited in the review as relevant references.

**Results and discussion:**

Pure compounds derived from African medicinal plants with demonstrated anti-malarial/antiplasmodial properties with activities ranging from “very active” to “weakly active” have been discussed. The majority of the 187 natural products were terpenoids (30%), followed by flavonoids (22%), alkaloids (19%) and quinones (15%), with each of the other compound classes being less than 5% of the entire compound collection. It was also observed that most of the plant species from which the compounds were identified were of the families Rubiaceae, Meliaceae and Asphodelaceae. The review is intended to continue laying the groundwork for an African-based anti-malarial drug discovery project.

## Background

Malaria is an endemic disease in most tropical countries (Africa, Asia, and Latin America), with about half of the world’s population at risk of infection according to the World Health Organization (WHO) [1]. According to the latest World Malaria Report, released in December 2019, there were 228 million cases of malaria in 2018, and the estimated number of malaria deaths stood at 405,000. The causative agents for malaria infections are *Plasmodium* protozoans (i.e. *Plasmodium falciparum*, *Plasmodium malariae*, *Plasmodium ovale*, and *Plasmodium vivax*), although most severe infections are caused by *P. falciparum* [2–4]. Most deaths are recorded among African children below the age of 5 years [1–4]. This calls for an urgent need for new anti-malarial therapies for any one of the following reasons:

- The development of resistance against insecticides (e.g. dichlorodiphenyltrichloroethane, DDT) by the disease vectors (female anopheline mosquitoes) [5–7].
- The inefficacy of chemoprophylaxis, which has often resulted in poor results [1, 8–10].
- The development of resistance by *Plasmodium* protozoans against most of the drugs currently used to treat malaria (e.g. chloroquine, artemisinin and its derivatives) [11–13].

Plants are known to be a rich reservoir of bioactive secondary metabolites (or natural products, NPs), for example, the anti-malarial drugs quinine and artemisinin (AT) are both of plant origin [14]. The benefits of plants containing bioactive anti-malarial compounds, particularly the bitter principles (alkaloids and terpenoids), include their use in the preparation of traditional remedies against malaria, fever, and inflammation [15]. In fact, more than 80% of the local populations of most tropical countries, including African populations, are dependent on medicinal plants for the treatment of most diseases, including malaria, despite the current wide availability of standard malaria treatments for populations in the rural areas, as well as those in cities [16, 17]. It has become of interest to summarize the major findings regarding the most promising secondary metabolites with proven in vitro and in vivo potencies, so as to pave the way for further development with compounds from African sources as leads for anti-malarial drug discovery. Recent reviews have either emphasized plants used in specific countries or regions for the treatment of malaria [18–21], secondary metabolites from selected plant species [22] or families of species [23], plants used as repellents against the mosquito vectors [24], or reports on the analysis of components of improved traditional preparations against malaria [25, 26].

Previous reviews have described the in vitro and in vivo potencies of compounds that have been isolated from African floral matter published data before 2013 [27, 28]. These reviews had previously described over 500 NPs, within the major NP classes, including alkaloids, terpenoids, flavonoids, coumarins, phenolics, polyacetylenes, xanthones, quinones, steroids, and lignans. These compounds were described in the literature as exhibiting from weak to very good in vitro anti-malarial activities, based on well-established cut-off values [29–31]. Besides, a cheminformatic analysis of the aforementioned dataset, with a focus on molecular descriptors related to “drug-likeness”, drug metabolism and pharmacokinetics (DMPK), and some rules of thumb such as the Lipinski “Rule of Five” [32], showed that over 50% of the anti-malarial compounds had physicochemical properties that fell within the range of “drug-like” molecules [33].

The present review focuses on compounds with tested activities against various malaria parasites derived from a literature survey from 2013 to 2019 [29–31]. A total of 187 NPs belonging to diverse classes, including alkaloids, flavonoids, phenolics, flavonoids, steroids, and terpenoids are described. These compounds have been identified from 45 plant species belonging to 23 families. It is hoped that the results summarized will help for lead compound identification and for further anti-malarial drug discovery. The review describes the NPs with potential anti-malarial properties from African medicinal plants, arranged alphabetically according to the main NP compound classes.

## Materials and methods

### Data collection

In this review, an attempt has been made to document the anti-malarial activities of NPs derived from African medicinal plants. The data was based on the current version of the African compound libraries [34–37], which are constantly being updated based on inputs from journal articles and student theses (M.Sc/Ph.D.) available in African University libraries. Emphasis was laid on data published after 2012. The original data collection, now being included in the African Compounds Database (http://www.african-compounds.org), was conducted from querying individual journal websites using the country names in Africa and search terms. The list of journals visited have been included in Additional file 1. The “hit” articles were retrieved, i.e. those for which plant materials were collected from Africa were then hand-picked by reading through the “Materials and methods“ section to ensure that the plant materials were from Africa. Student theses were also randomly collected as made available from university libraries, constituting a small portion of the data. The folder containing the retained articles/theses was further queried with the search terms “malaria”, “malarial”, “plasmodium”, “plasmodial” and a combination of them. Those for which compounds further showed anti-malarial activities published between 2013 and 2019 and for which the measured activities fell within the established cut-off values were selected and included as relevant references. The compounds displaying anti-malarial properties were classified according to their NP classes and source species of origin. This represented 187 compounds from 45 species belonging to 23 plant families.

### Data analysis

The collected data was arranged into spreadsheets according to plant sources, compound classes, activity cut-offs and plasmodial strains tested. All activity data was converted to IC_50_ values in μM.

Throughout the text, the term antiplasmodial is referred to as that which counters the growth of parasites of the genus *Plasmodium*, while anti-malarial is referred to as an agent which prevents or counteracts the progress of the disease caused by the parasite or that which treats the disease (i.e. by killing the parasites in the host). Very often the two terms are used interchangeably in the literature surveyed.

### Test methodologies

From the literature collected, a broad range plasmodial strains were tested, including those summarized in Table 1.

**Table 1:** Summary of testing methodologies and parasite strains described in this report

Murine (in vivo) models	Strains	Parasite name	Origin	Assay description	References
CQ-sensitive	NK 173	*Plasmodium berghei*	Not reported	Not reported	
	ANKA	*P. berghei*	Not reported	Not reported	[38–40]
		*Plasmodium vinckei petteri*	Not reported	Not reported	
***In vitro models***					
CQ-sensitive	3D7	*P. falciparum*	Not reported	Parasite lactate dehydrogenase (pLDH) assay	[41, 42]
			Not reported	Parasite growth inhibition assay	[43]
			Not reported	Translation inhibitory assay	[44]
	D6	*P. falciparum*	Sierra Leone	Incorporated G-^3^H hypoxanthine assay	[41, 42, 45, 46]
				Parasite lactate dehydrogenase (pLDH) assay	[47, 48]
				Non-radioactive Malaria SYBR Green I assay	[49, 50]
				Modified non-radioactive Malaria SYBR Green I assay	[42, 49, 51]
	D10	*P. falciparum*	Not reported	pLDH assay	[42, 52]
	F_32_	*P. falciparum*	Tanzania	Not reported	
	FCA20	*P. falciparum*	Ghana	Not reported	
	K1	*P. falciparum*	Thailand	Modified [^3^H]-hypoxanthine incorporation assay and [^3^H]-hypoxanthine incorporation assay	[53–56]
	NF54	*P. falciparum*	Not reported	[^3^H]-hypoxanthine incorporation assay	[53–56]
CQ-resistant	Dd2	*P. falciparum*	Not reported	Non-radioactive Malaria SYBR Green I assay	[49, 50]
	FcM_29_	*P. falciparum*	Cameroon	Not reported	
	FcB1	*P. falciparum*	Colombia	[^3^H]-hypoxanthine incorporation assay	[41]
	K1	*P. falciparum*	Thailand	Modified [^3^H]-hypoxanthine incorporation assay and [^3^H]-hypoxanthine incorporation assay	[53–56]
	W2	*P. falciparum*	Indochina	Modified non-radioactive Malaria SYBR Green I assay	[42, 49, 51]
				Incorporated G-^3^H hypoxanthine assay	[45, 46]
				Non-radioactive Malaria SYBR Green I assay	[49, 50]
	NF54	*P. falciparum*		[^3^H]-hypoxanthine incorporation assay	[41]
CQ- and pyrimethamine-resistant	K1	*P. falciparum*	Thailand	Modified [^3^H]-hypoxanthine incorporation assay and [^3^H]-hypoxanthine incorporation assay	[53–55]
	NF54	*P. falciparum*	Thailand	[^3^H]-hypoxanthine incorporation assay	[54]
Multidrug-resistant	Dd2	*P. falciparum*	Not reported	[^3^H]-hypoxanthine incorporation assay	[53–56]
	K1	*P. falciparum*	Thailand	Parasite lactate dehydrogenase (pLDH) assay	[52, 57]
	NF54	*P. falciparum*	Not reported	[^3^H]-hypoxanthine incorporation assay	[53–56]
	W2	*P. falciparum*	Indochina	[^3^H]-hypoxanthine incorporation assay	[45, 46]
	W2mef	*P. falciparum*	Not reported	[^3^H]-hypoxanthine incorporation assay	[53–56]

### Promising anti-malarial compounds derived from the African flora

#### Alkaloids

**Fig. 1:**
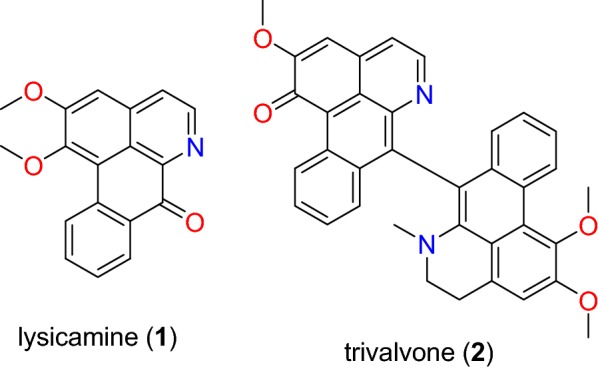
Aporphine alkaloids (**1** and **2**)

**Fig. 2:**
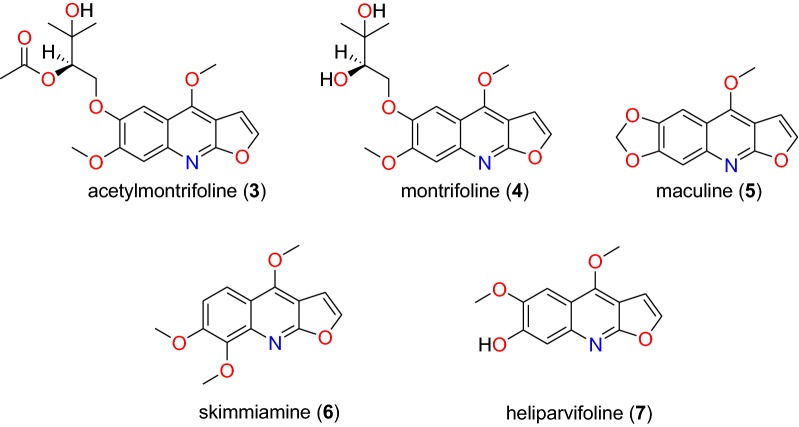
Furoquinoline alkaloids (**3** to **7**)

**Fig. 3:**
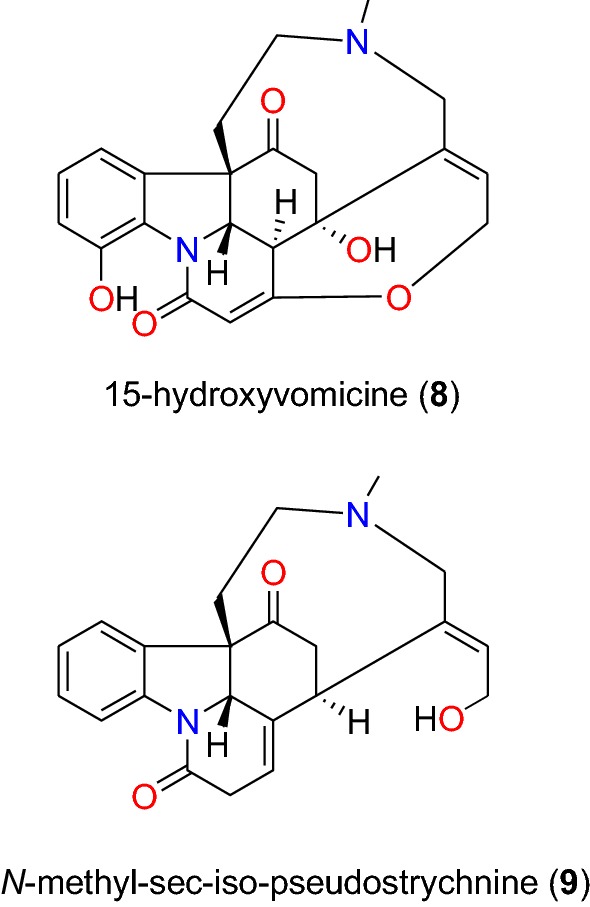
Indole alkaloids (**8** and **9**)

**Fig. 4:**
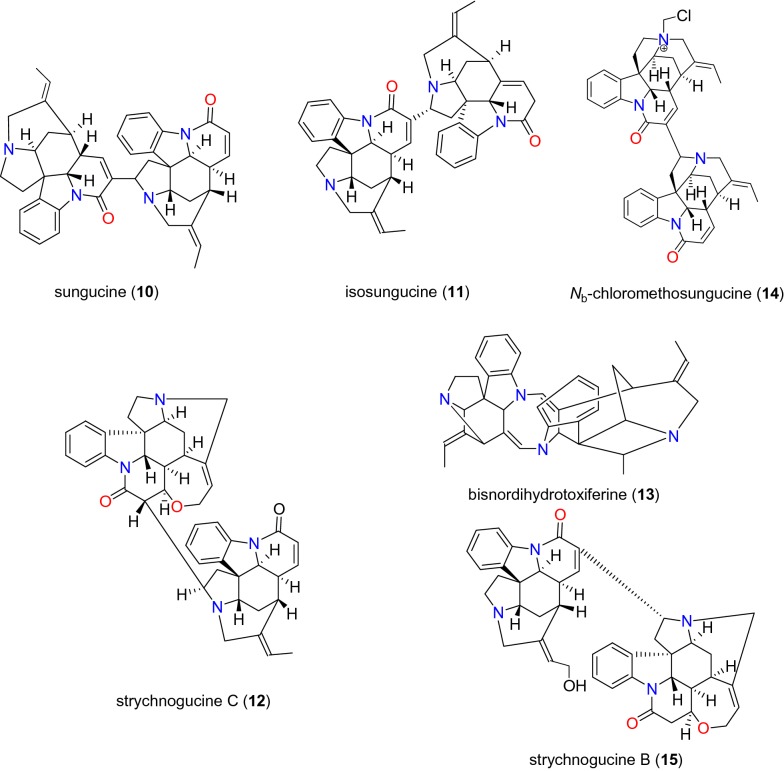
Indole alkaloids (**10** to **15**)

**Fig. 5:**
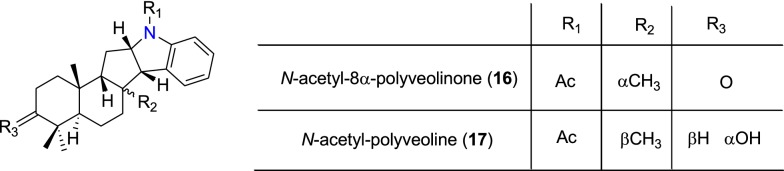
Indolosesquiterpene alkaloids (**16** and **17**)

**Fig. 6:**
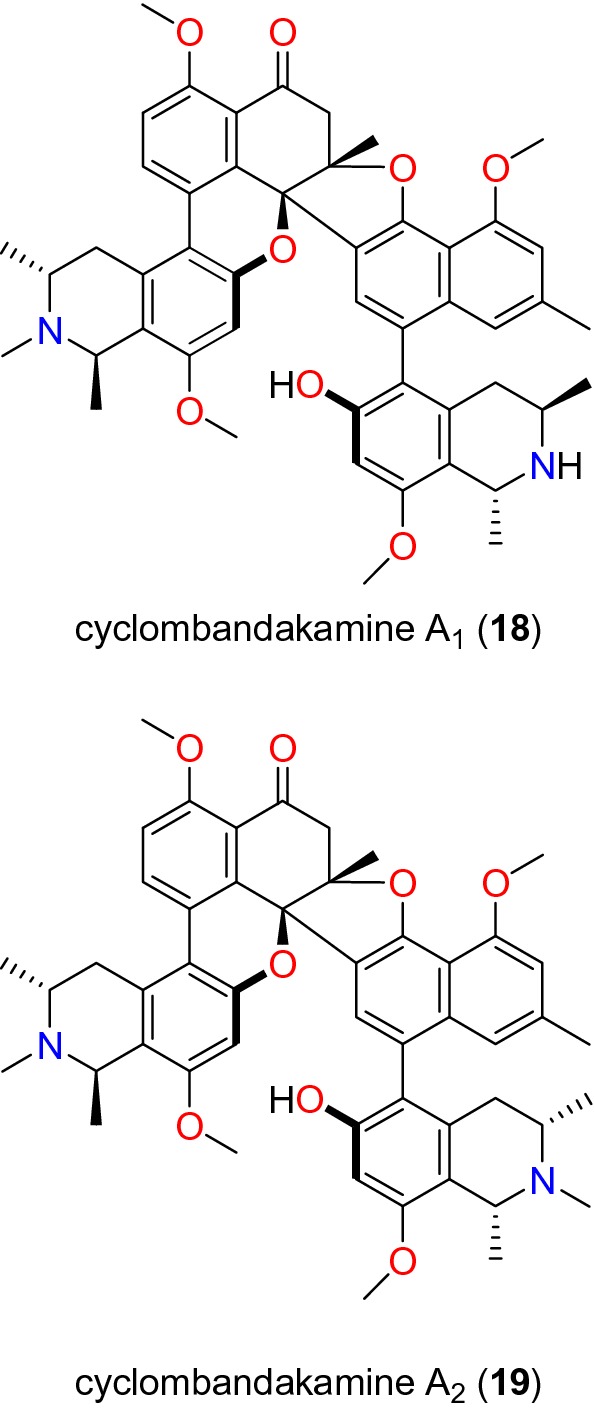
Naphthylisoquinoline alkaloids I (**18** and **19**)

**Fig. 7:**
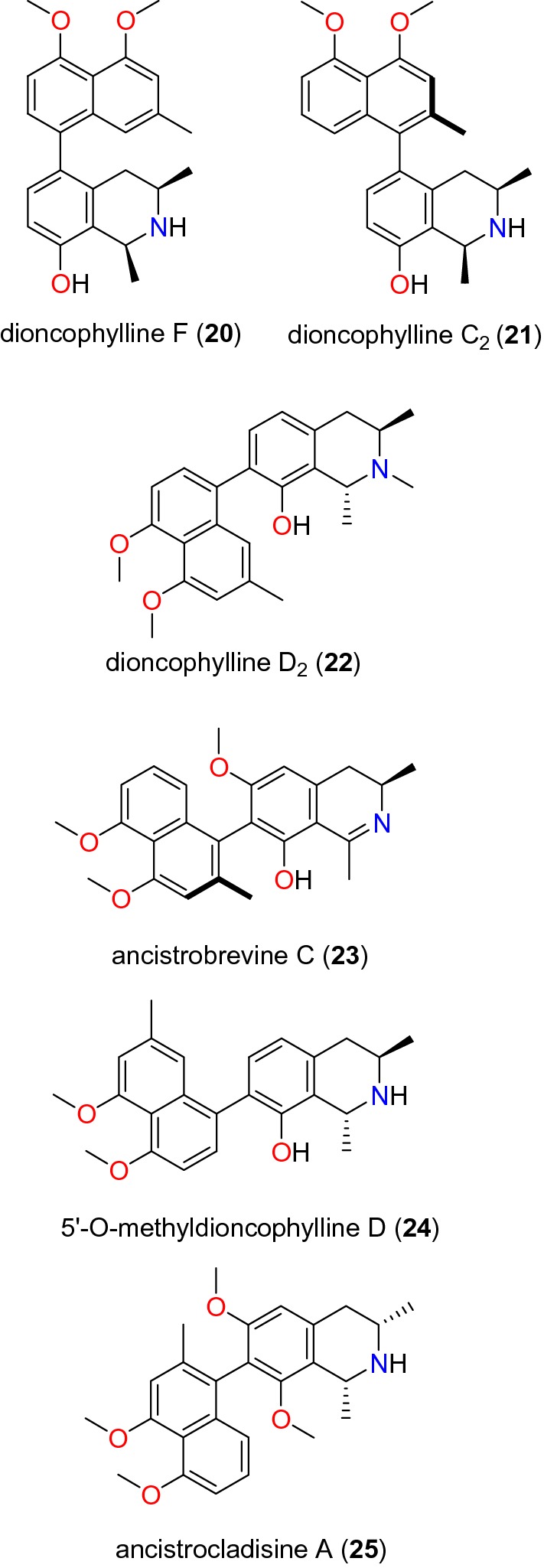
Naphthylisoquinoline alkaloids II (**20** to **25**)

**Fig. 8:**
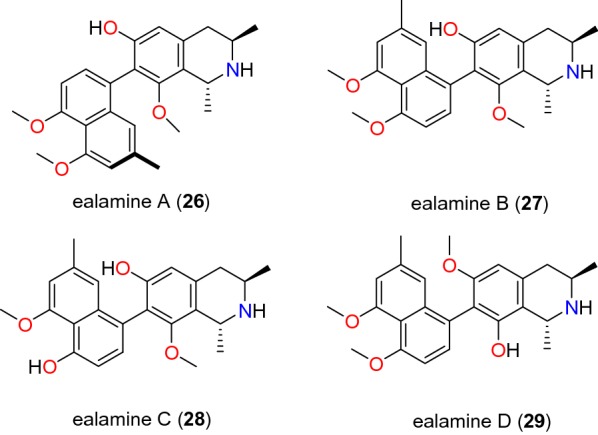
Naphthylisoquinoline alkaloids III (**26** to **29**)

**Fig. 9:**
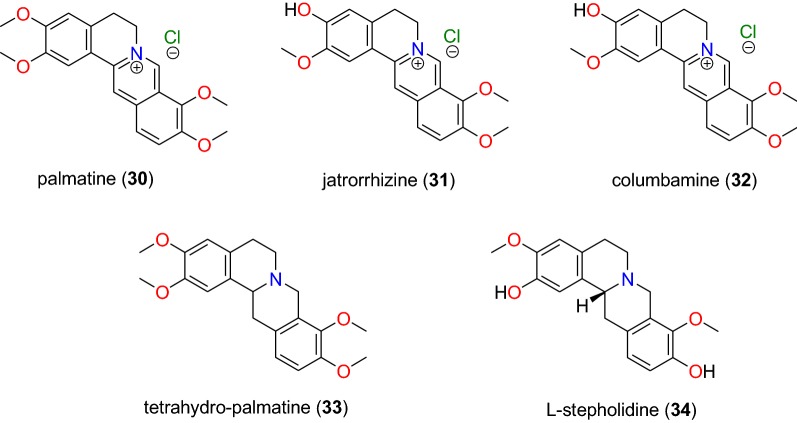
Protoberberine alkaloids (**30** to **34**)

**Fig. 10:**
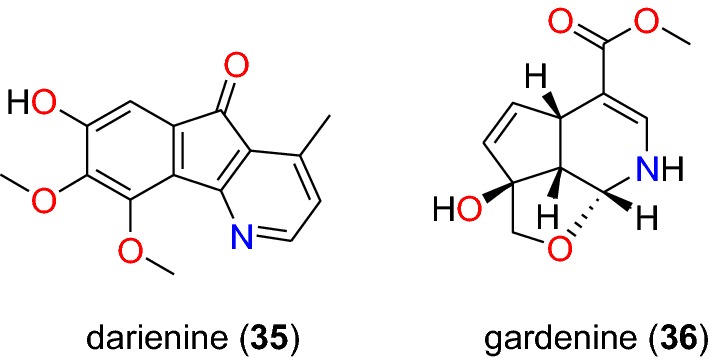
Other alkaloids (**35** and **36**)

Table 2 summarizes the most promising alkaloids derived from the African flora, published since the earlier review [27], while the chemical structures are shown in Figs. 1, 2, 3, 4, 5, 6, 7, 8, 9 and 10, arranged alphabetically according to their respective sub-classes (i.e. aporphines, furoquinolines, indoles, indolosesquiterpenes, Naphthylisoquinolines, protoberberines, pyridinones and others). Several of them had tested positive against CQ-sensitive and CQ-resistant strains of *P. falciparum* in vitro.

**Table 2:** Summary of alkaloids

Compound subclass	Isolated metabolites	Plasmodial strain (activities)	Plant species (Family), Taxon ID^b^	Part of the plant studied	Place of harvest (Locality, Country)	Author, references
Aporphines	**1** to **2**	K1 (8.24 and 2.90 μM, respectively)	*Annickia kummeriae* (Annonaceae), NCBI:txid225831	Leaves	Amani Nature Reserve, Tanzania	Malebo et al. [58]
Furoquinolines	**3**^a^ to **6**	FcB1 (from 162.47 to 298.16 μM)	*Teclea nobilis* (Rutaceae), NCBI:txid1220089	Fruits and leaves	Kamwenge district, Uganda	Lacroix et al. [59]
	**7**	Dd2 (IC_50_ = 35 μM)	*Melicope madagascariensis* (Rutaceae), NCBI:txid1487113	Stem bark	Antsasaka forest of Moramanga, Madagascar	Rasamison et al. [60]
Indoles	**8**^a^ to **13** and **15**	3D7 (from 0.41 to 110.58 μM)	*Strychnos icaja* (Loganiaceae), NCBI:txid1040889	Stem bark	Bertoua, Cameroon	Tchinda et al. [61]
	**14**	FCA20 (0.617 μM)	*Strychnos icaja* (Loganiaceae), NCBI:txid1040889	Roots	Kasongo-Lunda, DR Congo	Beaufay et al. [62], Frédérich et al. [63]
		W2 (0.085 μM)				
Indolosesquiterpenes	**16**^a^ and **17**^a^	NF54 (7.6 μM and 29.1 μM, respectively)	*Polyalthia oliveri* (Annonaceae), NCBI:txid105756	Stem bark	Mount Kala, Cameroon	Kouam et al. [64]
Naphthylisoquinolines	**18**^a^ and **19**^a^	NF54 (0.043 and 0.055 μM, respectively)	*Ancistrocladus* sp. (Ancistrocladaceae), NCBI:txid 63071	Leaves	Mbandaka, DR Congo	Lombe et al. [65]
	**20**^a^, **21`**^a^, **22**^a^ and **23** to **25**	NF54 (from 0.090 to 6.54 μM)	*Ancistrocladus ileboensis* (Ancistrocladaceae), NCBI:txid1367080	Leaves and root bark	Bambange, DR Congo	Li et al. [66]
		K1 (0.228 μM for compound **19**)				
	**26**^a^, **27**^a^, **28**^a^ and **29**^a^	NF54 (from 0.84 to 22.2 μM)	*Ancistrocladus ealaensis* (Ancistrocladaceae), NCBI:txid714098	Twigs and leaves	Mbandaka, DR Congo	Tshitenge et al. [67]
		K1 (from 1.4 to 8.2 μM)				
Protoberberines	**30** to **33**	K1 (from 0.22 to 0.71 μM)	*Annickia kummeriae* (Annonaceae), NCBI:txid225831	Leaves	Amani Nature Reserve, Tanzania	Malebo et al. [58]
	**34**	K1 (IC_50_ = 318.66 μM)	*Polyalthia longifolium* var. *pendula* (Annonaceae), NCBI:txid235806	Stem	Tikrom, near Kumasi, Ghana	Gbedema et al. [68]
Pyridinones	**35**	K1 (IC_50_ = 81.28 μM)	*Polyalthia longifolium* var. *pendula* (Annonaceae), NCBI:txid235806	Stem	Tikrom, near Kumasi, Ghana	Gbedema et al. [68]
Others	**36**	K1 (IC_50_ = 32.12 μM)	*Canthium multiflorum* (Rubiaceae), NCBI:txid58501	Aerial part	Obala, along River Sanaga, Cameroon	Kouam et al. [69]

^a^Compounds identified for the first time in the cited publications

^b^Identification number of the source species, derived from the NCBI Taxonomy database

##### Aporphines

The aporphine alkaloids lysicamine (**1**), trivalvone (**2**) (Fig. 1) were identified from the leaves of *Annickia kummeriae* (Annonaceae) from Tanzania, along with four other (protoberberine) alkaloids. Plants from the genus *Annickia* (formerly *Enantia*) are popularly known in West and Central Africa for use in the treatment of malaria [70–72]. The study by Maleba et al. [58] showed that compounds **1** and **2** showed respective activities of 8.23 and 2.90 μM against the CQ-resistant K1 strain of *P. falciparum*.

##### Furoquinolines

Four furoquinoline alkaloids (**3** to **6**) (Fig. 2) were isolated from the fruits and leaves of *Teclea nobilis* (Rutaceae) and tested on the chloroquine (CQ)-resistant FcB1/Colombia strain of *P. falciparum* by Lacroix et al. [59]. This species from Uganda has been used to treat a range of ailments from pain and fever to malaria [73]. The isolated compounds, including the novel acetylmontrifoline (**3**), and the known montrifoline (**4**), maculine (**5**), and skimmianine (**6**), were less potent than the reference drug CQ, showing inhibition against the tested parasite strain at < 300 μM [59].

Rasamison et al. also isolated seven furoquinolines from the stem bark of the Madagascan species, *Melicope madagascariensis* (Rutaceae), of which only compound **7** (6-methoxy-7-hydroxydictamnine, commonly called heliparvifoline) exhibited weak anti-malarial activity against the CQ-resistant strain, Dd2, with IC_50_ = 35 μM, the other compounds tested being inactive [60].

##### Indoles

*Strychnos icaja* (Loganiaceae) is found all over Central Africa [74]. In Cameroon, for example, the roots are used by a Pygmy tribe to treat malaria. From their stem bark, six indole alkaloids (**8** to **13**) (Figs. 3 and 4), were isolated and evaluated against the CQ-sensitive 3D7 strain of *P. falciparum* by Tchinda et al. [61], with IC_50_ values ranging from 0.40 to 110 μM. These include 15-hydroxyvomicine (**8**), *N*-methyl-sec-iso-pseudostrychnine (**9**), sungucine (**10**), isosungucine (**11**), strychnogucine C (**12**), bisnordihydrotoxiferine (**13**), along with the chlorinated indole, *N*_b_-chloromethosungucine (**14**).

Strychnogucine B (**15**) (Fig. 4), which was previously isolated from the roots of the same species by Frédérich et al. [63] was further investigated by Beaufay et al. [62] and the compound now displayed further inhibition against the CQ-sensitive FCA 20/Ghana and CQ-resistant W2/Indochina strains, with IC_50_ values of 0.617 and 0.085 μM, respectively.

##### Indolosesquiterpenes

The bioactivity-guided screening of the stem bark of *Polyalthia oliveri* (Annonaceae) led Kouam et al. to isolate two indolosesquiterpene alkaloids, named *N*-acetyl-8α-polyveolinone (**16**) and *N*-acetyl-polyveoline (**17**) (Fig. 5) [64]. This species is used in folk medicine for the treatment of malaria [75]. Both compounds were tested against CQ-sensitive NF54 strain and compound **16** showed moderate antiplasmodial activity with IC_50_ = 7.6 μM, while compound **17** inhibited the strain weakly with an IC_50_ value of 29.1 μM [75].

##### Naphthylisoquinolines

These are compounds characterized by a chiral biarylaxis linkage between the naphthalene and the isoquinoline alkaloids, mainly isolated from plants of the genus *Ancistrocladus* (Acistrocladaceae), and the closely related genera *Triphyophyllum*, *Dioncophyllum* and *Habropetalum* (Dioncophyllaceae). Cyclombandakamines A_1_ (**18**) and A_2_ (**19**) (Fig. 6) are naphthoisoquinoline alkaloids isolated from the leaves of *Ancistrocladus* sp. (Ancistrocladaceae) by Lombe et al. [65]. These compounds displayed significant inhibitory activities against the NF54 strain of *P. falciparum* with IC_50_ values of 0.043 and 0.055 μM, respectively [65]. Li et al. [66] also investigated the twigs and leaves of *Ancistrocladus ileboensis* (Ancistrocladaceae) from DR Congo. Among the tested compounds with promising anti-malarial activities were dioncophylline F (**20**) and dioncophylline C_2_ (**21**), in addition to the 7,8′-coupled dioncophylline D_2_ (**22**), ancistrobrevine C (**23**), 5′-*O*-methyldioncophylline D (**24**), and ancistrocladisine A (**25**) (Fig. 7). Compound **20** showed activities against both the NF54 and K1 strains (with IC_50_ values of 0.090 and 0.045 μM, respectively), compounds **21** to **25** were only tested against the K1 strain, with IC_50_ values ranging from 0.107 to 6.51 μM [66].

Among the compounds identified from Ancistrocladaceae, Tshitenge et al. also isolated four naphthylisoquinolines, named ealamines A–D (**26** to **29**, Fig. 8) from the twigs and leaves of *Ancistrocladus ealaensis* (Ancistrocladaceae) harvested in Mbandaka, DR Congo [67]. These compounds were tested against CQ-sensitive NF54 and CQ- and pyrimethamine-resistant K1 strains of *P. falciparum*. The activities against the CQ-sensitive NF54 strain showed IC_50_ values of 6.3, 4.9, 0.84 and 22.2 μM, respectively. Meanwhile, compounds **26**, **27** and **29** inhibited the CQ- and pyrimethamine-resistant K1 strain with IC_50_ values of 1.6, 1.4, and 8.2 μM, respectively [67].

##### Protoberberines

Maleba et al. [58] showed that against the CQ-resistant K1 strain of *P. falciparum* protoberberine alkaloids are a subclass of promising anti-malarials. The in vitro testing of compounds **30** to **33** (Fig. 9) showed that compound **30** (palmatine) was the most active, with an IC_50_ value of 0.23 μM. Jatrorrhizine (**31**) exhibited an IC_50_ of 0.71 μM, whereas a mixture of compound **31** and columbamine (**32**) inhibited the plasmodial strain with an IC_50_ value of 0.14 μg/mL, and a mixture of compound **26** and tetrahydro-palmatine (**33**) inhibited the parasite strain with IC_50_ = 0.098 μg/mL, probably explaining the synergistic activity of this plant extract. This justifies its use in African Traditional Medicine for the treatment of malaria [58].

Extracts of *Polyalthia longifolium* (Annonaceae), used in orally consumed preparations in traditional medicine in Ghana, was investigated in order to identify anti-malarial compounds [68]. The protoberberine l-stepholidine (**34**, Fig. 9) was identified from the stem of species among the isolated compounds [68], but this compound had only a weak antiplasmodial activity against the K1 strain of *P. falciparum*.

##### Pyridinones

Gbedema et al. also isolated darienine (**35**, Fig. 10), a known alkaloid with anti-malarial activity [68]. This compound exhibited varying degrees of antiplasmodial activity against the K1 strain of *P. falciparum* with an IC_50_ value of 81.28 μM.

##### Other alkaloids

Gardenine (**36**, Fig. 10), obtained from the investigation of crude extract of the aerial parts of *Canthium multiflorum* (Rubiaceae), harvested from Cameroon, exhibited antiplasmodial activity against the K1 strain of *P. falciparum*, with an IC_50_ value of 32.12 μM and weak cytotoxicity against L6 cell lines [69].

#### Flavonoids

**Fig. 11:**
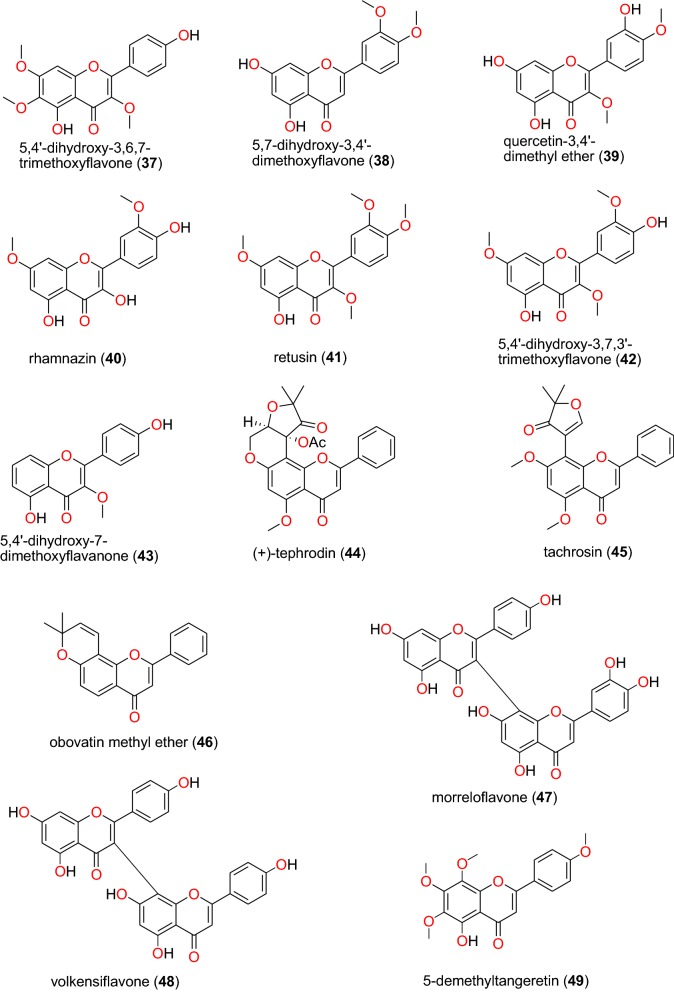
Flavanones and flavones (**37** to **49**)

**Fig. 12:**
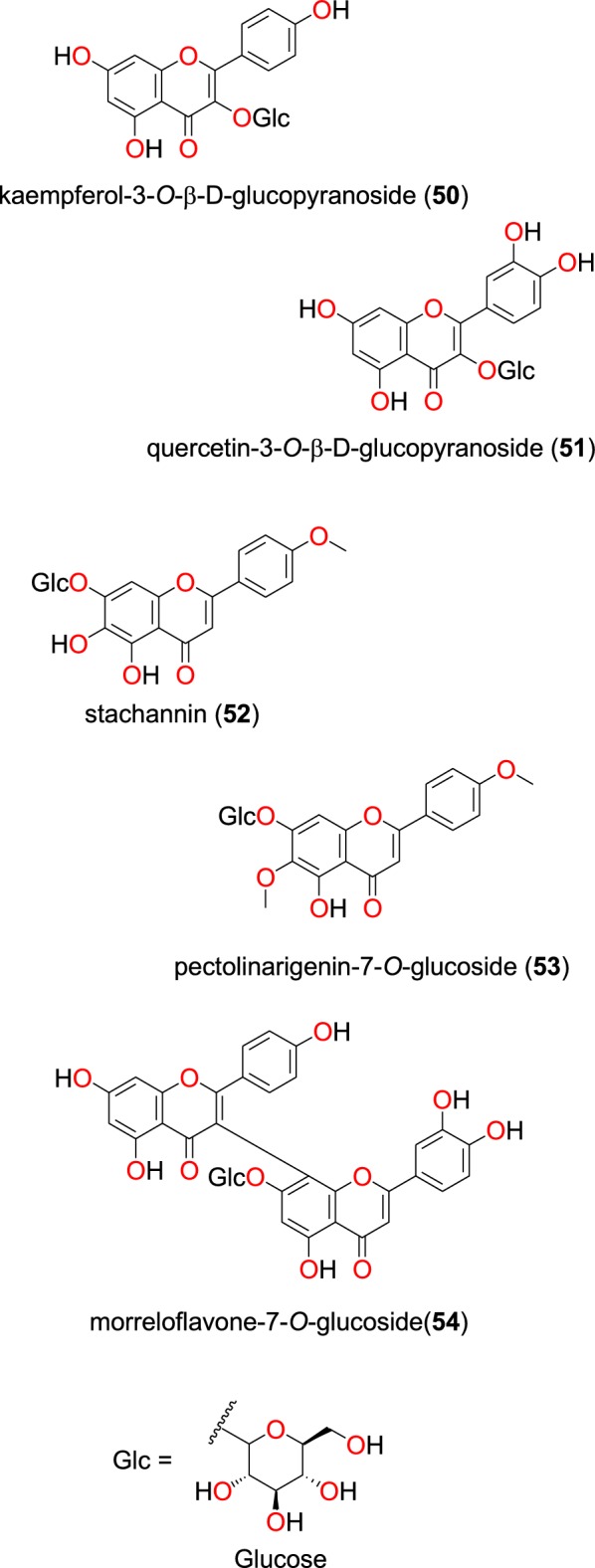
Glycoflavonoids (**50** to **54**)

**Fig. 13:**
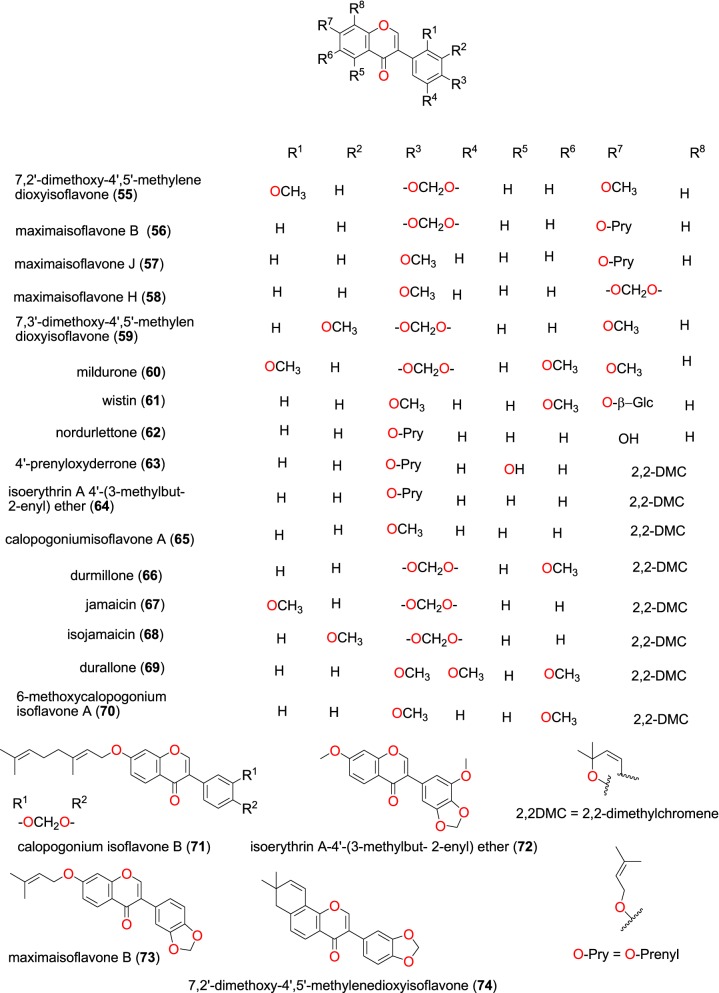
Isoflavones (**55** to **74**)

**Fig. 14:**
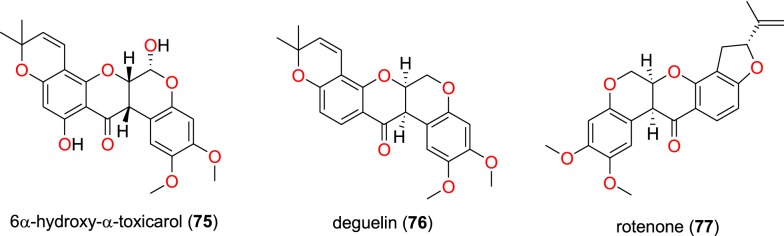
Retonoids (**75** to **77**)

Flavonoids (mainly chalcone, flavanone, isoflavone, and retonoid sub-classes) (Figs. 11, 12, 13 and 14) were previously seen as a promising class of NPs exhibiting anti-malarial and antiplasmodial activities [28].

##### Flavanones and flavones

The most promising recently published anti-malarial flavanones and flavones derived from the African flora have been summarized in Table 3. These include 5,4′-dihydroxy-3,6,7-trimethoxyflavone (**37**), 5,7-dihydroxy-3,4′-dimethoxyflavone (**38**), quercetin-3,4′-dimethyl ether (**39**), rhamnazin (**40**), retusin (**41**), 5,4′-dihydroxy-3,7,3′-trimethoxyflavone (**42**), 5,4′-dihydroxy-7-dimethoxyflavanone (**43**), (+)-tephrodin (**44**), tachrosin (**45**), obovatin methyl ether (**46**), morreloflavone (**47**), volkensiflavone (**48**), and 5-demethyltangeretin (**49**), whose chemical structures of are shown in Fig. 11. The compounds were isolated from the species *Senecio roseiflorus* (Compositae-Asteraceae) [76], *Tephrosia villosa* (Leguminosae) [77], *Allanblackia floribunda* (Guttiferae) [78], and *Peperomia vulcanica* (Piperaceae) [79].

**Table 3:** Summary of flavonoids

Compound subclass	Isolated metabolites	Plasmodial strain (activities)	Plant species (Family), Taxon ID^b^	Part of the plant studied	Place of harvest (locality, country)	Author, references
Flavanones and flavones	**37** to **43**	D6 (from 11.26 to 56.31 μM)	*Senecio roseiflorus* (Compositae-Asteraceae), NCBI:txid1886451	Leaves	Mount Kenya Forest, Meru, Kenya	Kerubo et al. [76]
		W2 (from 15.48 to 87.50 μM)				
	**44** to **46**	D6 (from 11.30 to 14.00 μM)	*Tephrosia villosa* (Leguminosae-Fabaceae), NCBI:txid62125	Roots	Manyani, TaitaTaveta County, Kenya	Muiva-Mutisya et al. [77]
		W2 (from 13.10 to 20.40 µM)				
	**47** and **48**	F32 (from 2.18 to 21.13 μM)	*Allanblackia floribunda* (Guttiferae- Clusiaceae), NCBI:txid469914	Whole plant	Mount Kala, Cameroon	Azebaze et al. [78]
		FcM29 (from 1.75 to 22.59 μM)				
	**49**	W2mef (19.37 μM)	*Peperomia vulcanica* (Piperaceae), NCBI:txid1719589	Whole plant	Mount Cameroon, Cameroon	Ngemenya et al. [79]
		Dd2 (3.18 μM)				
Glycoflavonoids	**50** and **51**	D6 (97.1 and 42.9 μM, respectively)	*Ekebergia capensis* (Meliaceae), NCBI:txid124949	Leaves	Gakoe Forest, Kiambu County, Kenya	Irungu et al. [80]
		W2 (105.8 μM)				
	**52** and **53**		*Gardenia ternifolia* (Rubiaceae), NCBI:txid1237590; *Crossopteryx febrifuga* (Rubiaceae), NCBI:txid170354; and *Lantana camara* (Verbenaceae), NCBI:txid126435	Stem barks and leaves	Kinshasa, DR Congo	Tshitenge et al. [26]
	**54**	F32 (15.98 and 40.36 μM, respectively, at 24 h and 72 h)	*Allanblackia floribunda* (Guttiferae- Clusiaceae), NCBI:txid469914	Whole plant	Mount Kala, Cameroon	Azebaze et al. [78]
		FcM29 (11.69 and 33.24 µM , respectively, at 24 h and 72 h)				
Isoflavones	**55 to 70**	W2 (from 14.9 to 53.1 μM)	*Millettia oblata* ssp. *teitensis* (Leguminosae-Fabaceae), NCBI:txid53625	Stem bark	Taita Hill Forest, Kenya	Derese et al. [81]
	**63**^a^	D6 (from 13.3 to 48.7 μM)				
	**71**^a^ to **74**	3D7 and Dd2 (70 to 90% inhibition at 40 μM)	*Millettia dura* (Leguminosae-Fabaceae), NCBI:txid62119	Root bark	Kisarawe, Tanzania	Marco et al. [82]
Retonoids	**75** to **77**	D6 (18.71 µM for compound **75** and 9.60 μg/mL for a mixture of compounds **76** and **77**)	*Tephrosia villosa* (Leguminosae-Fabaceae), NCBI:txid62125	Roots	Manyani, Taita Taveta County, Kenya	Muiva-Mutisya et al. [77]
		W2 (28.64 µM for compound **75** and 22.60 μg/mL for a mixture of compounds **76** and **77**)				

^a^Compounds identified for the first time in the cited publications

^b^Identification number of the source species, derived from the NCBI Taxonomy database

Compounds **37** to **43** were derived from leaves of *Senecio roseiflorus* and have shown good to moderate antiplasmodial activities against D6 and W2 strains. The activities in terms of IC_50_ values ranged from 11.25 to 56.31 µM for the D6 strain, while for the W2 strain, this ranged from 15.47 to 87.50 µM [76]. Compounds **44** to **46**, were derived from roots of *Tephrosia villosa* and exhibited anti-malarial activities against both the D6 and W2 strains with respective IC_50_ values from 11.30 to 14.00 µM for the D6 strain and from 13.10 to 20.40 µM for the W2 strain [77].

Azebaze et al. investigated the antiplasmodial activities of whole plant extracts of *Allanblackia floribunda* from Cameroon [78]. The biflavonoids **47** and **48** were isolated from the plant extract and exhibited in vitro antiplasmodial activities against the F32 and FcM29 strains. The IC_50_ values at 24 h and 72 h against the both strains were 21.13 and 6.03 µM; 22.59 and 8.61 µM for compound **47** and 1.83 and 2.18 µM; 1.44 and 1.75 µM for compound **48**, respectively [78]. Additionally, 5-demethyltangeretin (**49**) was isolated from the whole plant of *Peperomia vulcanica* by Ngemenya et al. [79]. This compound showed antiplasmodial activity against the multidrug-resistant W2mef and Dd2 strains of *P. falciparum*, with respective IC_50_ values of 19.36 and 3.18 µM.

##### Glycoflavonoids

The glycoflavonoids kaempferol-3-*O*-β-d-glucopyranoside (**50**), and quercetin-3-*O*-β-d-glucopyranoside (**51**), Fig. 12, were isolated from the leaves of *Ekebergia capensis* (Meliaceae) from Kenya by Irungu et al. [80]. Both compounds were observed to possess moderate activities against the D6 and W2 strains of *P. falciparum*. The IC_50_ values of compounds **50** and **51** were, respectively, 97.1 and 42.9 µM against the D6 strain, while both compounds measured an IC_50_ value of 105.8 µM against the W2 strain.

Tshitenge et al. investigated the anti-malarial constituents of the medicinal plant-based SIROP KILMA, with constitutive plants composed from *Gardenia ternifolia* (Rubiaceae), *Crossopteryx febrifuga* (Rubiaceae), and *Lantana camara* (Verbenaceae) [26]. The authors identified two flavonoid glycosides; stachannin (**52**) and pectolinarigenin-7-*O*-glucoside (**53**) [26]. The flavone glycoside morreloflavone-7**-***O***-**glucoside (**54**) was isolated by Azebaze et al. from *Allanblackia floribunda* (Guttiferae), harvested in Cameroon. This compound presented antiplasmodial activities against the F32 and FcM29 strains with IC_50_ values of 15.98 and 11.69 µM; 40.36 and 33.24 µM at 24 h and 72 h, respectively [78].

##### Isoflavones

Studies by Derese et al. on the stem bark of *Millettia oblata* (Leguminosae) harvested from Kenya led to the isolation of 7,2′-dimethoxy-4′,5′-methylenedioxyisoflavone (**55**), maximaisoflavone B (**56**), maximaisoflavone J (**57**), maximaisoflavone H (**58**), 7,3′-dimethoxy-4′,5′-methylendioxyisoflavone (**59**), mildurone (**60**), wistin (**61**) nordurlettone (**62**), 4′-prenyloxyderrone (**63**), isoerythrin A 4′-(3-methylbut-2-enyl) ether (**64**), calopogoniumisoflavone A (**65**), durmillone (**66**), jamaicin (**67**), isojamaicin (**68**), durallone (**69**), and 6-methoxycalopogonium isoflavone A (**70**) (Fig. 13) [81]. The plant extracts and isolated compounds were tested in vitro against the *P. falciparum* W2 and D6 strains. All the plant extracts had IC_50_ values ranging from 10.0 to 25.4 μg/mL. The compounds showed good to moderate antiplasmodial activities with the following pairs of IC_50_ values; 45.6 and 47.5; 42.0 and 36.0; 29.7 and 35.7; 38.8 and 45.6; 48.4 and 37.7; 44.1 and 35.9; 23.2 and 22.3; 28.9 and 25.1; 14.9 and 13.3; 21.6 and 19.3; 51.5 and 45.8; 25.1 and 37.3; 38.6 and 41.0; 38.9 and 48.7; 50.0 and 32.7; 53.1 and 34.8 μM against the W2 and D6 strains, respectively [81].

Marco et al. obtained calopogonium isoflavone B (**71**) and isoerythrin A-4′-(3-methylbut-2-enyl) ether (**72**) maximaisoflavone B (**73**) and 7,2′-dimethoxy-4′,5′-methylenedioxyisoflavone (**74**) from the root bark of *Millettia dura* (Leguminosae) harvested in Tanzania [82]. These compounds showed marginal activities (70 to 90% inhibition at 40 μM) against the 3D7 and Dd2 strains of *P. falciparum*.

##### Retonoids

Muiva-Mutisya et al. also isolated the retonoids 6α-hydroxy-α-toxicarol (**75**), deguelin (**76**), rotenone (**77**) (Fig. 14), from the root extract of *Tephrosia villosa* (Leguminosae) [77]. The mixture of compounds **76** and **77** exhibited anti-malarial activities with IC_50_ values of 9.60 and 22.60 µg/mL against the CQ-sensitive D6 and CQ-resistant W2, respectively. Meanwhile, the activities of compound **75** against the same strains were 18.71 and 28.64 µM, respectively [77].

#### Phenolics and quinones

**Fig. 15:**
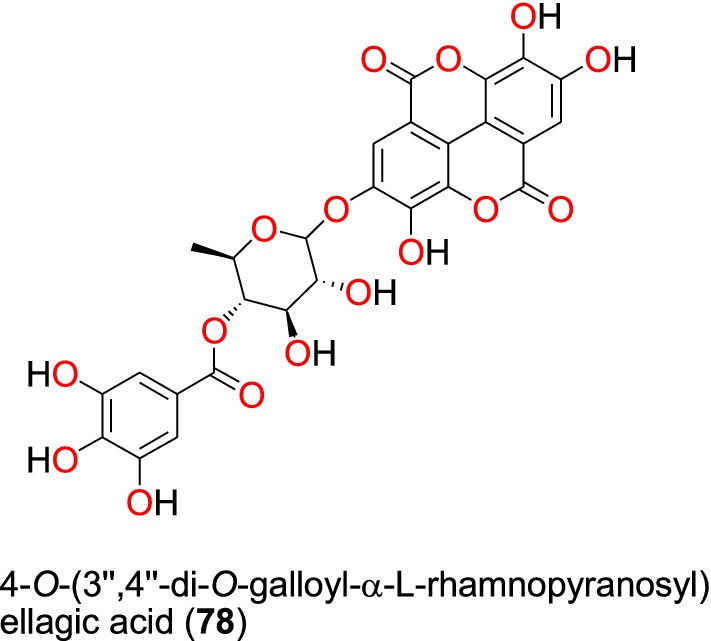
Ellagic acid derivative (**78**)

**Fig. 16:**
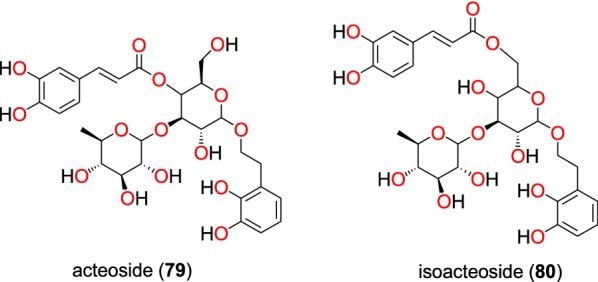
Phenolic glycosides (**79** and **80**)

**Fig. 17:**
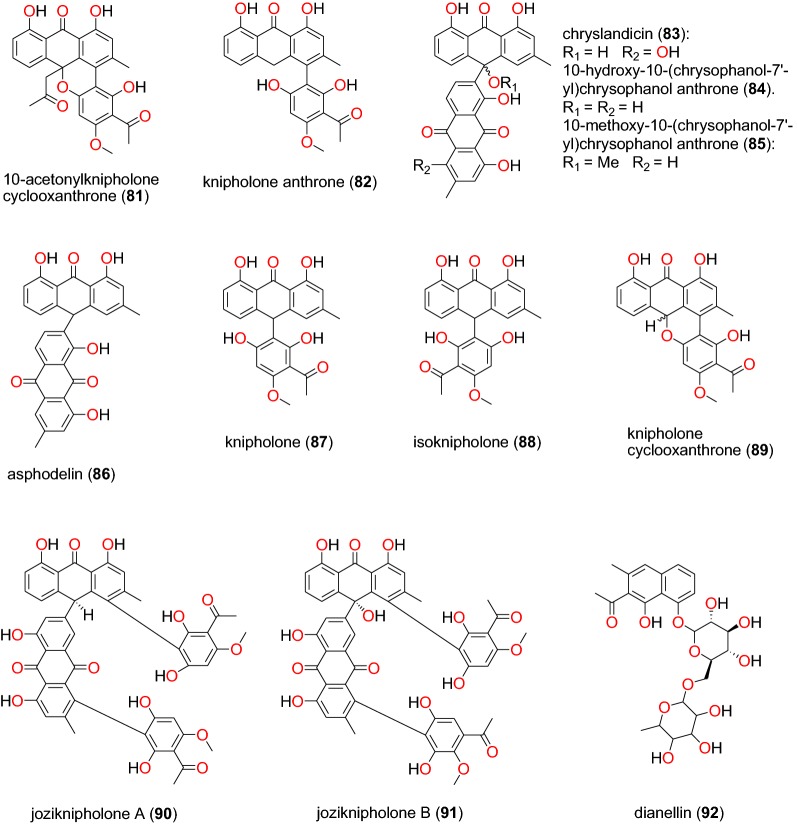
Anthraquinones (**81** to **92**)

**Fig. 18:**
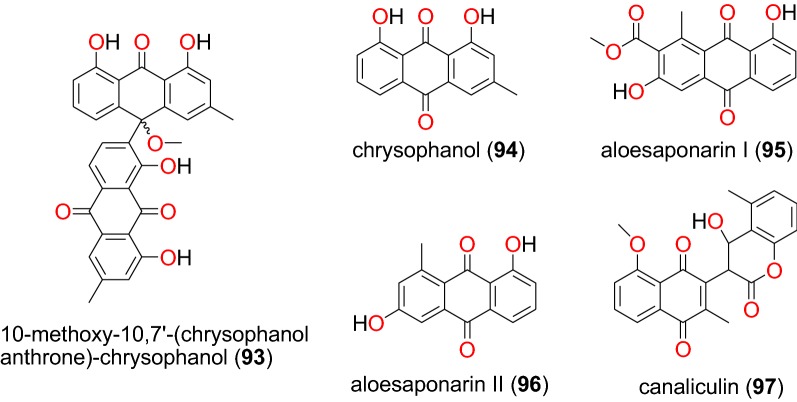
Anthraquinones (**93** to **97**)

**Fig. 19:**
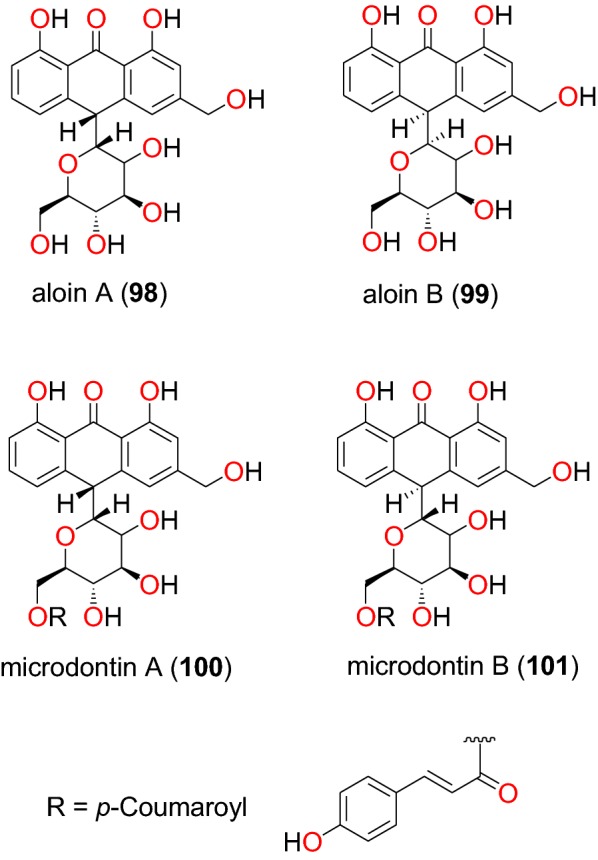
Anthrones (**98** to**101**)

**Fig. 20:**
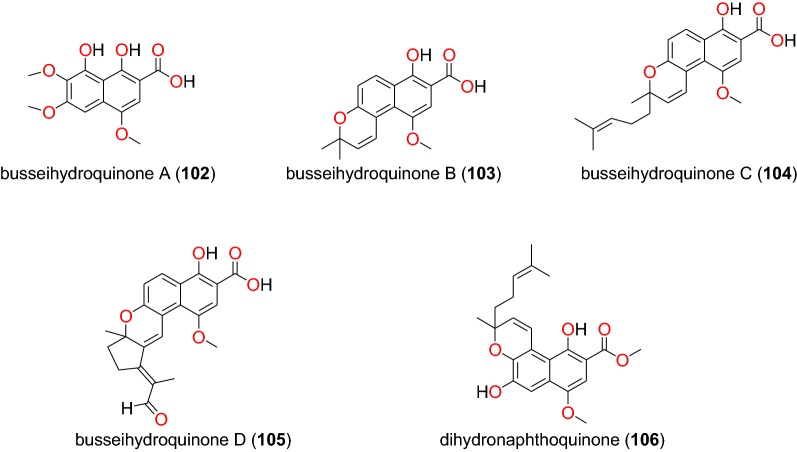
Naphthohydroquinones (**102** to **106**)

**Fig. 21:**
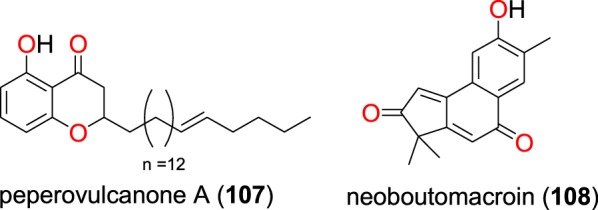
Other quinones (**107** and **108**)

Summaries of the phenolics and quinones with most promising anti-malarial properties have been shown in Table 4 (according to their subclasses), with chemical structures shown in Figs. 15, 16, 17, 18, 19, 20 and 21.

**Table 4:** Summary of phenolics and quinones

Compound subclass	Isolated metabolites	Plasmodial strain (activities)	Plant species (Family), Taxon ID^b^	Part of the plant studied	Place of harvest (locality, country)	Author, references
Ellagic acid derivative (phenolics)	**78**	D6 (8.01 µM)	*Terminalia brownii* (Combretaceae), NCBI:txid1548809	Stem bark	Machakos County, Kenya	Machumi et al. [84]
		W2 (8.01 µM)				
Phenolic glycosides (phenolics)	**79** and **80**		*Gardenia ternifolia* (Rubiaceae), NCBI:txid1237590; *Crossopteryx febrifuga* (Rubiaceae), NCBI:txid170354; and *Lantana camara* (Verbenaceae), NCBI:txid126435	Stem barks and leaves	Kinshasa, DR Congo	Tshitenge et al. [26]
Anthraquinones (quinones)	**81**^a^, **82** to **92**	D6 (from 0.47 to 23.25 μM)	*Kniphofia foliosa* (Asphodelaceae), NCBI:txid214838	Rhizomes	Addis Ababa, Ethiopia	Induli et al. [85]
	**89**	W2 (from 0.35 to 18.42 μM )				
	**93**	D6 (7.73 μM)	*Kniphofia foliosa* (Asphodelaceae), NCBI:txid214838	Roots	Gedo, Ethiopia	Abdissa et al. [86]
		W2 (2.22 μM)				
	**89**^a^	D6 (9.40 μM)	*Kniphofia foliosa* (Asphodelaceae), NCBI:txid214838	Roots	Gedo, Ethiopia	Abdissa et al. [86]
		W2 (14.58 μM)				
	**82**	3D7 (0.7 μM)	*Kniphofia foliosa* (Asphodelaceae), NCBI:txid214838	Leaves	Addis Ababa, Ethiopia	Feilcke et al. [89]
	**90**^a^ and **91**^a^	K1 (0.17 and 0.26 μm, respectively)	*Bulbine frutescens* (Asphodelaceae), NCBI:txid210954	Roots	Chiromo Campus Garden, Kenya	Bringmann et al. [87]
	**94** to **96**	D6 (19.66 to 82.80 μM)	*Aloe pulcherrima* (Asphodelaceae), NCBI:txid25641	Roots	Saka Chokorsa, Ethiopia	Abdissa et al. [88]
		W2 (64.46 to 141.95 μM)				
	**86**	3D7 (1.9 μM)	*Kniphofia foliosa* (Asphodelaceae), NCBI:txid214838	Leaves	Addis Ababa, Ethiopia	Feilcke et al. [89]
	**97**^a^	NF54 (weak activity)	*Diospyros canaliculata* (Ebenaceae), NCBI:txid13492	Stem bark	Kribi, Cameroon	Lenta et al. [90]
Anthrones (quinones)	**98** to **101**	Suppression of parasitaemia from 36.8 to 66.8% at doses of 100 to 400 mg/kg /day	*Aloe percrassa* (Asphodelaceae), NCBI:txid1593100	Leaf latex	Edagahamus, Ethiopia	Geremedhin et al. [91]
Naphthohydroquinones (quinones)	**102**^a^, **103**^a^, **104**^a^, **105**^a^ and **106**^a^	D6 (from 19.59 to 36.03 μM)	*Pentas bussei* (syn: *Rhodopentas bussei*, Rubiaceae), NCBI:txid387051	Roots	Mombasa, Kenya	Endale et al. [92]
		W2 (60.08 to 144.43 μM)				
Other quinones	**107**	W2mef (52.25 μM)	*Peperomia vulcanica* (Piperaceae), NCBI:txid1719589	Whole plant	Mount Cameroon, Cameroon	Ngemenya et al. [79]
	**108**^a^	D6 (19.28 μM)	*Neoboutonia macrocalyx* (Euphorbaceae), NCBI:txid316724	Stem bark	Kibale National Park, Uganda	Namukobe et al. [93]
		W2 (14.17 μM)				

^a^Compounds identified for the first time in the cited publications

^b^Identification number of the source species, derived from the NCBI Taxonomy database

##### Ellagic acid derivatives

The plant *Terminalia brownii* (Combretaceae) is used as a remedy for malaria in Eastern and Central Africa, although the detailed mode of preparation is not fully described in the literature [83]. The phenolic compound, 4**-***O***-**(3″,4″-di-*O*-galloyl-α-l-rhamnopyranosyl) ellagic acid (**78**) (Fig. 15), obtained from the stem bark of this plant harvested in Kenya was found to be active against chloroquine-sensitive (D6) and chloroquine-resistant (W2) strains of *P. falciparum* [77]. According to Machumi et al. [84], the IC_50_ value obtained against both strains was equal to 8.01 µM.

##### Phenolic glycosides

In addition to flavonoid glycosides, Tshitenge et al. [26] identified two phenolic glycosides as anti-malarial constituents of the medicinal plant-based SIROP KILMA: acteoside (**79**) and isoacteoside (**80**) (Fig. 16).

##### Anthraquinones

The study by Induli et al. of the rhizomes of *Kniphofia foliosa* (Asphodelaceae), growing in Ethiopia, led to the identification of several anthraquinones [85]: the novel 10-acetonylknipholone cyclooxanthrone (**81**), along with the known knipholone anthrone (**82**), chryslandicin (**83**), 10-hydroxy**-**10**-**(chrysophanol-7′-yl)-chrysophanol anthrone (**84**), 10-methoxy-10-(chrysophanol-7′-yl)chrysophanol anthrone (**85**), asphodelin (**86**), knipholone (**87**), isoknipholone (**88**) knipholone cyclooxanthrone (**89**), joziknipholone A (**90**), joziknipholone B (**91**) and dianellin (**92**) (Fig. 17). According to the authors, the IC_50_ values obtained for the plant extracts ranged from 3.4 to 8.9 μg/mL and from 3.4 to 8.9 μg/mL against the D6 and W2 strains of *P. falciparum*, respectively. The obtained compounds had IC_50_ values ranging from 0.47 to 23.25 μM and from 0.35 to 18.42 μM against the respective strains [85]. It is known that knipholone cyclooxanthrone (**89**) was actually isolated for the first time by Abdissa et al. and was also shown to exhibit antiplasmodial activities against the W2 and D6 strains with IC_50_ values of 14.58 and 9.42 μM, respectively [86].

The roots of the same plant, also harvested in Ethiopia, led Abdissa et al. to isolate a dimeric anthraquinone, 10-methoxy-10,7′-(chrysophanol anthrone)-chrysophanol (**93**) [86]. Compound **93** showed antiplasmodial activities against the W2 and D6 strains with IC_50_ values of 2.22 and 7.73 μM, respectively [86]. The investigations of Bringmann et al. led to the isolation of new dimeric phenylanthraquinones; joziknipholone A (**90**) and joziknipholone B (**91**) from the roots of *Bulbine frutescens* (Asphodelaceae) harvested in Kenya [87]. The authors also tested the two compounds against the K1 strain of *P. falciparum* and obtained remarkable activities, IC_50_ values of 0.17 and 0.26 μM, respectively [87]. Knipholone anthrone (**82**) was tested again from the leaves of the Ethiopian medicinal plant *Kniphofia foliosa* (Asphodelaceae) by Feilcke et al. [89]. The activity of this compound in several biological assays was described by the authors and showed antiplasmodial activity against 3D7 strain with IC_50_ value 0.7 μM.

The medicinal plant *Aloe pulcherrima* (Asphodelaceae) is one of the endemic *Aloe* species traditionally used for the treatment of malaria and wound healing in Central, Southern, and Northern Ethiopia, although the detailed mode of usage is not properly described in the literature [88]. Three compounds, chrysophanol (**94**), aloesaponarin I (**95**) and aloesaponarin II (**96**) (Fig. 18), were isolated from the acetone root extracts by Abdissa et al. [88]. The evaluation of their in vitro anti-malarial activities revealed moderate activity against D6 and W2 strains with IC_50_ values ranging from 19.66 to 82.80 μM and from 64.46 to 141.95 μM, respectively [88]. Knipholone (**86**) was also tested again from the leaves of *Kniphofia foliosa* (Asphodelaceae) by Feilcke et al. [89], showing significant antiplasmodial activity against the *P. falciparum* 3D7 strain, with an IC_50_ value of 1.9 μM.

Lenta et al. investigated the dichloromethane-methanol (1:1) extract of the stem bark of *Diospyros canaliculata* (Ebenaceae) harvested in Cameroon and obtained a new coumarinyl naphtoquinone, named canaliculin (**97**) [90]. The compound only exhibited weak activity against *P. falciparum* NF54 strain, unfortunately, along with pronounced toxicity [90].

##### Anthrones

The plant *Aloe percrassa* (Asphodelaceae), is an indigenous species used in Ethiopian folk medicine to treat malaria, wounds and gastric problems [91]. Aloin A (**98**) Aloin B (**99**) microdontin A (**100**) microdontin B (**101**) (Fig. 19), are four anthrones derived from the leaf latex of *Aloe percrassa* by Geremedhin et al. [91]. The anti-malarial activities of the mixtures of Aloin A/B and microdontin A/B were lower than the latex. The mixtures were shown to have suppressed parasitaemia from 36.8 to 66.8% at doses of 100 to 400 mg/kg/day. This suggested that the compounds within the two mixtures may have acted synergistically.

##### Naphthohydroquinones

The plant species *Pentas bussei* (Rubiaceae) is frequently used in traditional medicine to treat malaria in Kenya, particularly the boiling of the roots and stems for oral consumption [92]. The roots of this species led Endale et al. to obtain five new naphthohydroquinones, called busseihydroquinone A (**102**) busseihydroquinone B (**103**) busseihydroquinone C (**104**) busseihydroquinone D (**105**) and the homoprenylated naphthoquinone named dihydronaphthoquinone (**106**) (Fig. 20). These compounds exhibited marginal activities against the D6 and W2 strains with IC_50_ values ranged from 19.59 to 36.03 μM and from 60.08 to 144.43 μM, respectively [92].

##### Other quinones

Peperovulcanone A (**107**), derived from the crude extracts of the whole plant of *Peperomia vulcanica* (Piperaceae), harvested from Cameroon, was shown to be active against the W2mef strain of *P. falciparum* with an IC_50_ value of 52.25 μM [79]. The new compound named, neoboutomacroin (**108**), was derived from extracts of the stem bark of *Neoboutonia macrocalyx* (Euphorbiaceae) from Uganda by Namukobe et al. [93]. Compound **108** displayed good antiplasmodial activity with IC_50_ values of 19.28 and 14.17 μM against the D6 and W2 strains, respectively.

#### Steroids

##### Ergostane phytosterols

**Fig. 22:**
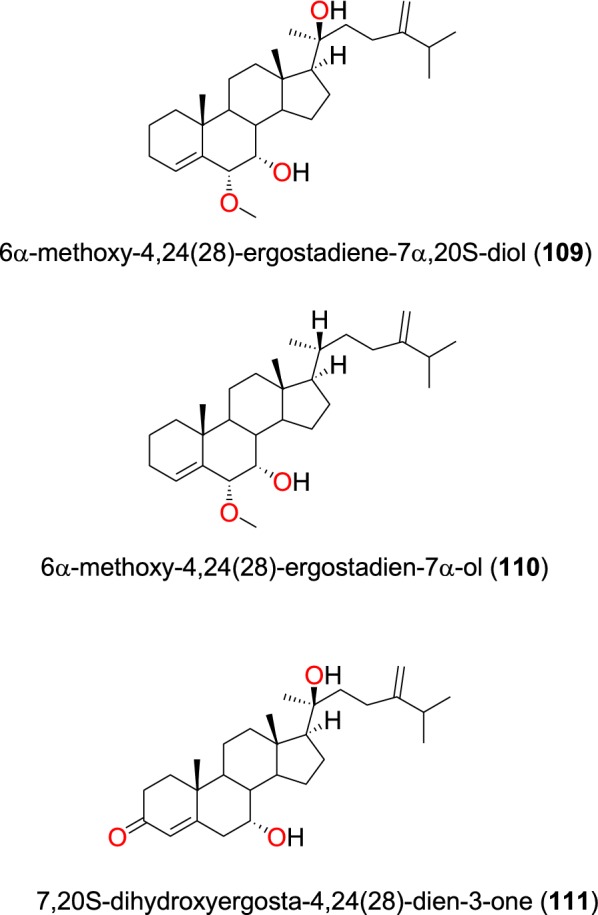
Ergostane phytosterols (**109** to **111**)

A summary of bioactive steroids has been provided in Table 5. The novel steroids; 6α-methoxy-4,24(28)-ergostadiene-7α,20S-diol (**109**), 6α-methoxy-4,24(28)-ergostadien-7α-ol (**110**) (Fig. 21), along with the known steroid 7,20*S*-dihydroxyergosta-4,24(28)-dien-3-one (**111**) (Fig. 22), were isolated from the stem bark of *Antrocaryon klaineanum* (Anacardiaceae) by Douanla et al. [94]. The crude extracts and the isolated compounds were evaluated in vitro against the 3D7 and W2 strains of *P. falciparum*. While the crude extract showed moderate activity (IC_50_ = 16.7 µg/mL) against 3D7, the three steroids exhibited potent activity against both strains with IC_50_ values of 22.0, 11.2 and 21.3 µM, respectively, against the same strain.

**Table 5:** Summary of steroids

Compound subclass	Isolated metabolites	Plasmodial strain (activities)	Plant species (Family), Taxon ID^b^	Part of the plant studied	Place of harvest (City, Country)	Author, references
Ergostane phytosterols	**109**^a^**, 110**^a^, and **111**	3D7 (IC_50_ values range from 11.2 to 22.0 µM)	*Antrocaryon klaineanum* (Anacardiaceae), NCBI:txid289695	Stem bark	Mount Kala, Cameroon	Douanla et al. [94]
		W2 ( IC_50_ values range from 11.2 to 22.0 µM)				
	**112**	W2mef (IC_50_ value = 53.45 µM)	*Peperomia vulcanica* (Piperaceae), NCBI:txid1719589	Whole plant	Mount Cameroon, Cameroon	Ngemenya et al. [79]
	**113**	W2 (IC_50_ value = 153.79 µM)	*Polyalthia longifolium* var. *pendula* (Annonaceae), NCBI:txid235806	Stem	Tikrom, near Kumasi, Ghana	Gbedema et al. [68]
	**113**	W2 (IC_50_ value = 172.9 µM)	*Turraea robusta* (Meliaceae), NCBI:txid1899148	Stem bark	Nairobi, Kenya	Irungu et al. [95]
		D6 (IC_50_ value = 68.3 µM)				
Phytosterol glucosides	**114** to **116**	D6 and W2 (from weak to moderate activities)	*Turraea nilotica* (Meliaceae), NCBI:txid992803	Stem bark	Nairobi, Kenya	Irungu et al. [95]

^a^Compounds identified for the first time in the cited publications

^b^Identification number of the source species, derived from the NCBI Taxonomy database

**Fig. 23:**
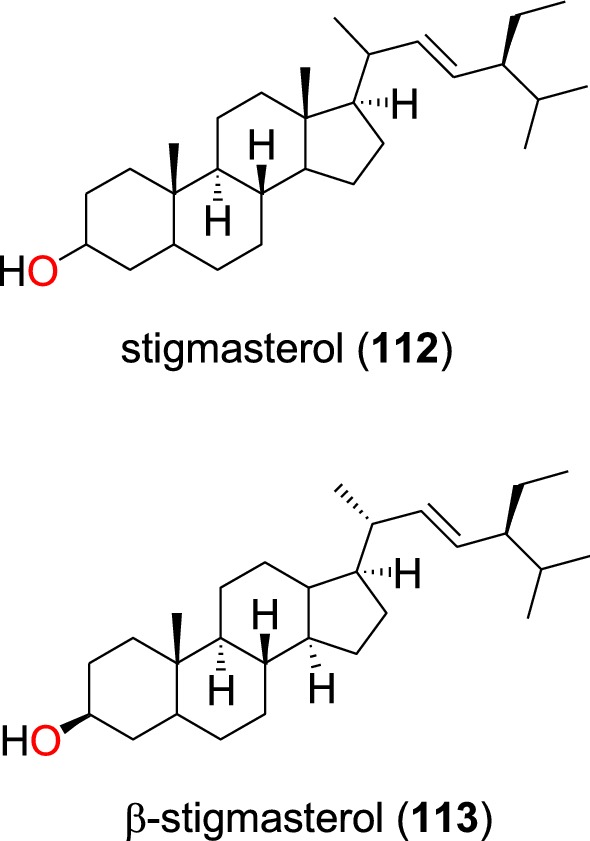
Ergostane phytosterols (**112** and **113**)

The known steroid stigmasterol (**112**) (Fig. 23), obtained from the whole plant of *Peperomia vulcanica* (Piperaceae), also showed antiplasmodial activity against the W2mef strain with an IC_50_ value of 53.45 µM [79]. β-stigmasterol (**113**) was also isolated from the stem of the *Polyalthia longifolium* (Annonaceae) harvested in Ghana [68]. This compound exhibited weak antiplasmodial activity against the K1, D6 and W2 strains of *P. falciparum* with IC_50_ values of 153.79, 68.3 and 172.9 µM, respectively [68, 94].

##### Phytosterol glucosides

**Fig. 24:**
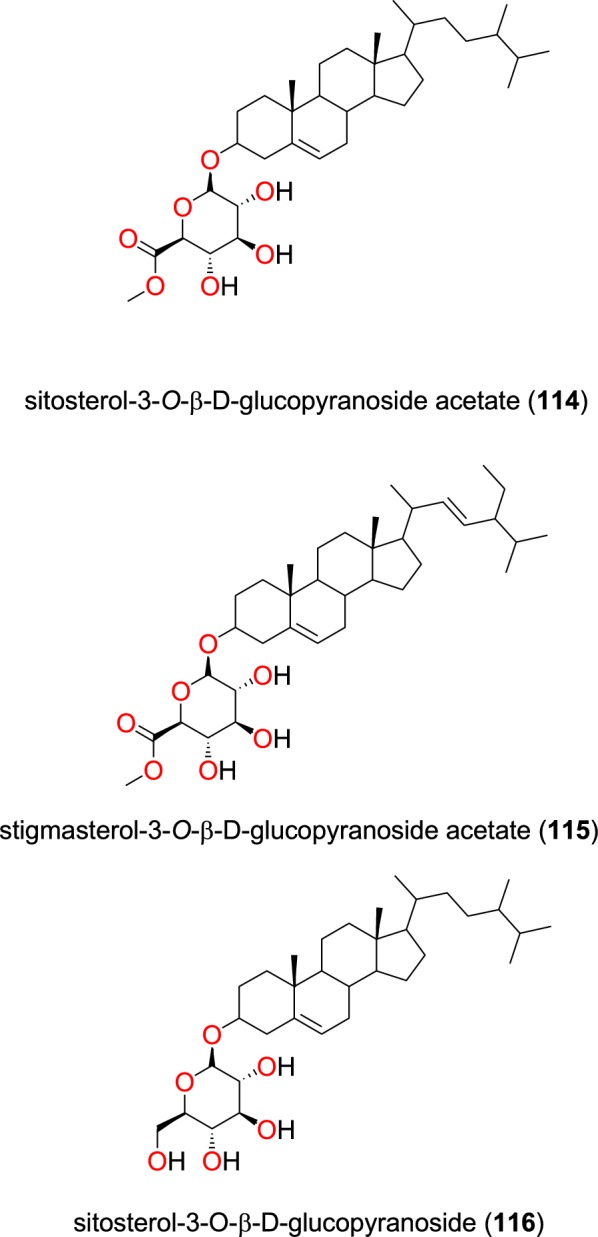
Phytosterol glucosides (**114** to **116**)

The known steroid glycosides; sitosterol-3-*O*-β-d-glucopyranoside acetate (**114**), stigmasterol-3-*O*-β-d-glucopyranoside acetate (**115**), sitosterol-3-*O*-β-d-glucopyranoside (**116**) (Fig. 24), as well as a mixture of β-sitosterol and stigmasterol (**112**) were identified from the leaves of *Turraea nilotica* (Meliaceae) [95]. The glycosides only showed weak to moderate antiplasmodial activities against the D6 and W2 strains.

#### Terpenoids

The summary of the most promising diterpenoids and sesquiterpenoids has been provided in Table 6, while those of triterpenoids have been shown in Table 7.

**Table 6:** Summary of diterpenoids and sesquiterpenoids

Compound subclass	Isolated metabolites	Plasmodial strain (activities)	Plant species (Family), Taxon ID^b^	Part of the plant studied	Place of harvest (Locality, Country)	Author, references
Clerodane diterpenes	**117** to **119**	K1 (IC_50_ values range from 9.59 to 18.41 µM)	*Polyalthia longifolium* var. *pendula* (Annonaceae), NCBI:txid235806	Stem	Tikrom, near Kumasi, Ghana	Gbedema et al. [68]
Daphnane diterpenoids	**120**	FcB1 (IC_50_ value = 19.02 µM)	*Neoboutonia macrocalyx* (Euphorbaceae), NCBI:txid316724	Stem bark	Kibale National Park, Uganda	Namukobe et al. [96]
	**121** and **122**	D6 (IC_50_ values = 65.14 and 6.96 µM, respectively)	*Neoboutonia macrocalyx* (Euphorbaceae), NCBI:txid316724	Stem bark	Kibale National Park, Uganda	Namukobe et al. [93]
		W2 (IC_50_ values = 57.82 and 4.10 µM, respectively)				
Iridoid diterpenoid	**123**	K1 (IC_50_ value = 171.68 µM)	*Canthium multiflorum* (Rubiaceae), NCBI:txid58501	Aerial part	Obala, along River Sanaga, Cameroon	Kouam et al. [69]
Labdane diterpenoids	**124**	Suppression of *Plasmodium berghei* at doses of 25, 50 and 100 mg/kg with chemosuppression values of 50.13, 65.58 and 73.16%, respectively.	*Otostegia integrifolia* (syn: *Rydingia integrifolia*, Lamiaceae), NCBI:txid483857	Leaves	Chancho, Central Ethiopia	Endale et al. [97]
Norcassane furanoditerpene	**125**	3D7 (IC_50_ value = 2.20 μM)	*Caesalpinia bonducella* (Caesalpiniaceae), NCBI:txid53845	Roots	Dar es Salaam Region, Tanzania	Nondo et al. [98]
		Dd2 (IC_50_ value = 4.16 μM)				
Sesquiterpenoids	**126**^a^, **127**^a^, **128**^a^, **129**^a^, and **130**^a^	W2 (IC_50_ values range from 1.71 to 2.63 µM)	*Salacia longipes* (Celastraceae), NCBI:txid662028	Seeds	Mount Kala, Cameroon	Mba’ning et al. [99]
	**131**^a^	NF54 (IC_50_ value = 15.69 μM)	*Scleria striatinux* (Cyperaceae), NCBI:txid1916803	Rhizomes	Oku, Cameroon	Nyongbela et al. [100]
		K1 (IC_50_ value = 13.54 μM)				

^a^Compounds identified for the first time in the cited publications

^b^Identification number of the source species, derived from the NCBI Taxonomy database

**Table 7:** Summary of triterpenoids

Compound subclass	Isolated metabolites	Plasmodial strain (activities)	Plant species (Family), Taxon ID^b^	Part of the plant studied	Place of harvest (Locality, Country)	Author, references
Acyclic triterpenes	**132** and **133**	D6 (IC_50_ values = 27.1 and 56.1 µM, respectively)	*Ekebergia capensis* (Meliaceae), NCBI:txid124949	Leaves	Gakoe forest, Kiambu County, Kenya	Irungu et al. [80]
		W2 (IC_50_ values = 66.9 and 64.3 µM, respectively)				
Apotirucallane triterpenoids	**134**^a^, **135**^a^, **136**^a^, **137**^a^, **138**^a^, **139**^a^, and **140** to **142**	NF54 (IC_50_ values range from 0.67 to 19.3 µM)	*Entandrophragma congoense* (Meliaceae), NCBI:txid2590899	Bark	Nkomokui, Cameroon	Happi et al. [101]
Cycloartane triterpenes	**143** to **150**	FcB1 (all IC_50_ values < 11 μM, the lowest value being 1.48 μM)	*Neoboutonia macrocalyx* (Euphorbaceae), NCBI:txid316724	Stem bark	Kibale National Park, Uganda	Namukobe et al. [96]
	^a^All new					
Lanostane triterpene	**151**^a^	D6 (IC_50_ value = 257.8 nM)	*Ganoderma* sp. (Ganodermataceae), NCBI:txid5314	Whole organism	Egypt	Wahba et al. [102]
		W2 (IC_50_ value = 2000.0 nM)				
Limonoids	**152**	D6 (IC_50_ value = 84.7 µM)	*Ekebergia capensis* (Meliaceae), NCBI:txid124949	Leaves	Gakoe forest, Kiambu County, Kenya	Irungu et al. [80]
		W2 (IC_50_ value = 150.2 µM)				
	**153** to **157**	D6 (IC_50_ values range from 2.4 to 36.6 µM)	*Turraea robusta* (Meliaceae), NCBI:txid1899148	Root bark	Nairobi, Kenya	Irungu et al. [95]
		W2 (from 1.1 to 40.5 µM)				
Oleanane triterpenes	**158** to **161**	D6 (IC_50_ values range from 38.8 to 205.0 µM)	*Ekebergia capensis* (Meliaceae), NCBI:txid124949	Leaves	Gakoe forest, Kiambu County, Kenya	Irungu et al. [80]
		W2 (IC_50_ values range from 76.7 to 179.4 µM)				
	**160** and **162**	3D7 (IC_50_ values = 59.4 and 32.4 µM, respectively)	*Keetia leucantha* (Rubiaceae), NCBI:txid 43504	Twigs	Adjarra-Ouémé, Benin Republic	Bero et al. [103]
	**162**, **163** and **164**	D10 (IC_50_ values range from 3.81 to 15.54 μM)	*Mimusops caffra* (Sapotaceae), NCBI:txid362720	Leaves	Durban, KwaZulu-Natal Province, South Africa	Simelane et al. [104]
Tirucallane-type triterpenoids	**165**^a^ , **166**^a^ and **167**	NF54 (IC_50_ values range from 2.4 to 6.1 µM)	*Entandrophragma congoense* (Meliaceae), NCBI:txid2590899	Bark	Nkomokui, Cameroon	Happi et al. [105]
Protolimonoids	**168** to **170**	D6 (IC_50_ values range from 36.8 to 48.2 µM)	*Turraea nilotica* (Meliaceae), NCBI:txid992803	Stem bark	Nairobi, Kenya	Irungu et al. [95]
		W2 (IC_50_ values range from 37.2 to 77.0 µM)				
Other triterpenoids (hopane-type and cycloartane-type)	**171**	NF54 (IC_50_ value = 112.94 μM)	*Diospyros canaliculata* (Ebenaceae), NCBI:txid13492	Stem bark	Kribi, Cameroon	Lenta et al. [90]
	**172**	NF54 (IC_50_ value = 97.73 μM)	*Erythrina caffra* (Papilionaceae), NCBI:txid3842	Stem bark	Pietermaritzburg, South Africa	Chukwujekwu et al. [106]
	**173**	FcB1( IC_50_ value = 2.15 µM)	*Neoboutonia macrocalyx* (Euphorbaceae), NCBI:txid316724	Stem bark	Kibale National Park, Uganda	Namukobe et al. [96]

^a^Compounds identified for the first time in the cited publications

^b^Identification number of the source species, derived from the NCBI Taxonomy database

##### Clerodane diterpenes

**Fig. 25:**
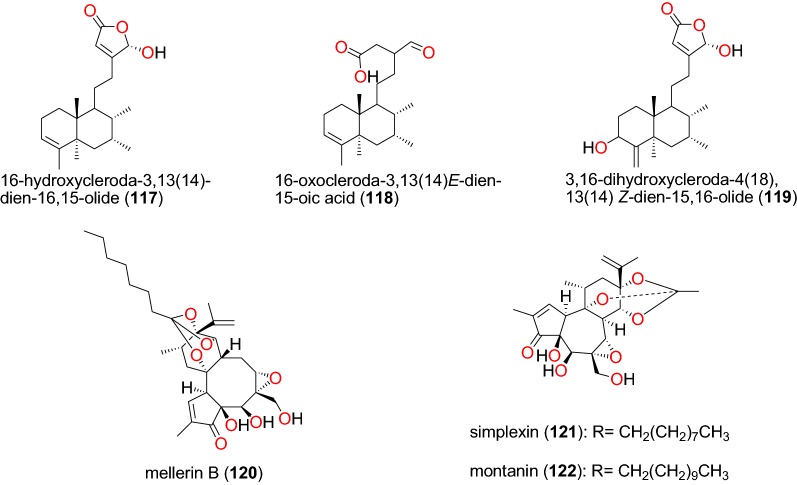
Clerodane and daphnane diterpenoids (**117** to **122**)

The ethanolic extract of *Polyalthia longifolium* var. *pendula*, which is traditionally used to treat malaria in Ghana (the traditional preparation not properly described in the literature) displayed in vitro antiplasmodial activity against the multidrug-resistant, K1 strain with an IC_50_ value of 22.04 μg/mL. Spectroscopic analysis of compounds obtained from this extract led to the identification of three known clerodane diterpenes; 16-hydroxycleroda-3,13(14)-dien-16,15-olide (**117**), 16-oxocleroda-3,13(14)*E*-dien-15-oic acid (**118**), and 3,16-dihydroxycleroda-4(18),13(14) *Z*-dien-15,16-olide (**119**) (Fig. 25) [68]. The compounds showed activities with IC_50_ values varying from 9.59 to 18.41 µM.

##### Daphnane diterpenes

The daphnane diterpenoid mellerin B (**120**) was isolated from the stem bark of *Neoboutonia macrocalyx* (Euphorbaceae) and potently inhibited the CQ-resistant FcB1/Colombia strain of *P. falciparum*, with an IC_50_ value 19.02 µM [96]. Chemical investigation of the stem bark of *Neoboutonia macrocalyx* (Euphorbiaceae) also yielded simplexin (**121**) and montanin (**122**) (Fig. 25), which showed antiplasmodial activities against the D6 and W2 strains, with IC_50_ values of 65.14 and 57.82 µM, respectively, and 6.96 and 4.10 µM, respectively [93].

##### Iridoids, labdanes, and norcassane furanoditerpenes

**Fig. 26:**
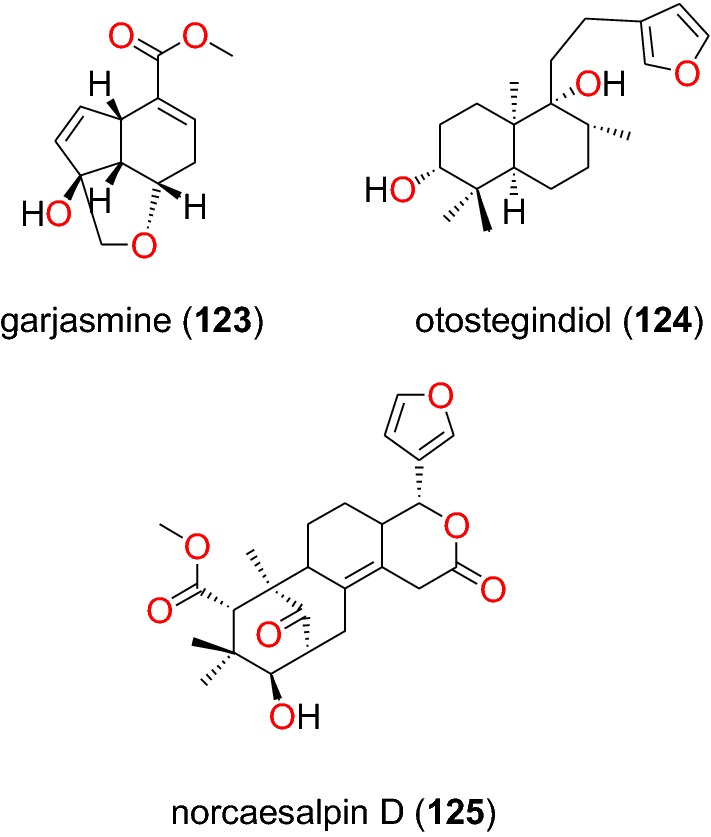
Iridoid, labdanes and norcassane diterpenoids (**123** to **125**)

From the aerial part of *Canthium multiflorum* (Rubiaceae) harvested in Cameroon, Kouam et al. also isolated the known iridoid, garjasmine (**123**) (Fig. 26) [69]. This compound only showed weak inhibition against the K1 strain of *P. falciparum*, with an IC_50_ value of 171.68 µM [69].

The leaves of *Otostegia integrifolia* (Lamiaceae) are used in Ethiopian folk medicine for the treatment of several diseases including malaria [97]. The known labdane diterpenoid, otostegindiol (**124**) (Fig. 26) was isolated from the methanol leaf extract of the species by Endale et al. [97]. The isolated compound 125 displayed a significant (p < 0.001) anti-malarial activity at doses of 25, 50 and 100 mg/kg with chemosuppression values of 50.13, 65.58 and 73.16%, respectively. The previously reported norcassane furanoditerpene, norcaesalpin D (**125**), was isolated from the roots of *Caesalpinia bonducella* (Caesalpiniaceae) from Tanzania by Nondo et al. [98]. This compound was active with an IC_50_ value of 2.20 and 4.16 µM against the 3D7 and Dd2 strains, respectively [98].

##### Sesquiterpenoids

**Fig. 27:**
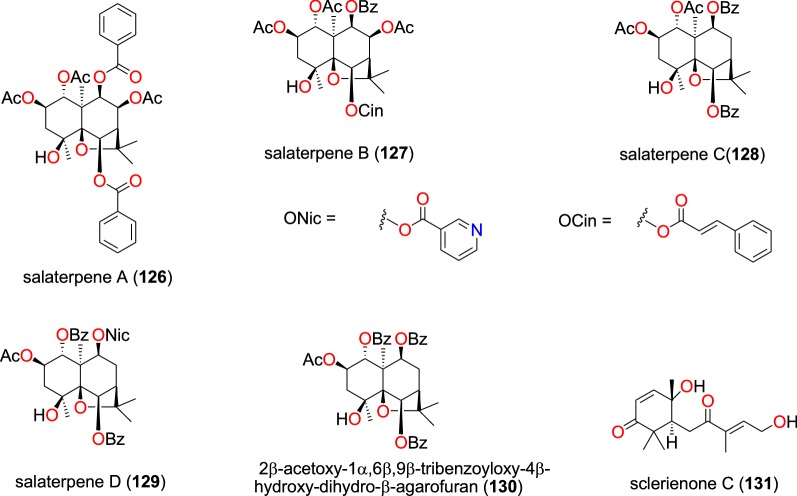
Sesquiterpenes (**126** to **131**)

The novel sesquiterpenoids salaterpenes A–D (**126** to **129**), and 2β-acetoxy-1α,6β,9β-tribenzoyloxy-4β-hydroxy-dihydro-β-agarofuran (**130**) (Fig. 27), were isolated from the seeds of *Salacia longipes* (Celastraceae), harvested in Cameroon by Mba’ning et al. [99]. The investigation of their potential for anti-malarial drug discovery demonstrated that these compounds inhibited the W2 strain of *P. falciparum* with IC_50_ values varying from 1.71 to 2.63 µM [99].

Nyongbela et al. [100] isolated the new sesquiterpene sclerienone C (**131**) from the rhizomes of *Scleria striatonux* (Cyperaceae), harvested from Cameroon. According to the authors, this compound exhibited antimicrobial and antiplasmodial activities with IC_50_ values against the NF54 and K1 strains of 15.69 and 13.54 μM, respectively [100].

##### Acyclic triterpenes

**Fig. 28:**
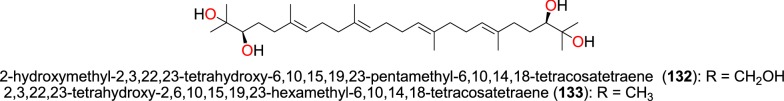
Acyclic triterpenes (**132** and **133**)

The previously reported acyclic triterpenes; 2-hydroxymethyl-2,3,22,23-tetrahydroxy-6,10,15,19,23-pentamethyl-6,10,14,18-tetracosatetraene (**132**) and 2,3,22,23-tetrahydroxy-2,6,10,15,19,23-hexamethyl-6,10,14,18-tetracosatetraene (**133**) were isolated from the leaves of the *Ekebergia capensis* (Meliaceae) harvested in Kenya [80]. The compounds (Fig. 28) exhibited selective antiplasmodial activity against the W2 strain, with IC_50_ values of 27.1 and 56.1 µM, and against the D6 66.9 and 64.3 µM, respectively [80].

##### Apotirucallane triterpenoids

**Fig. 29:**
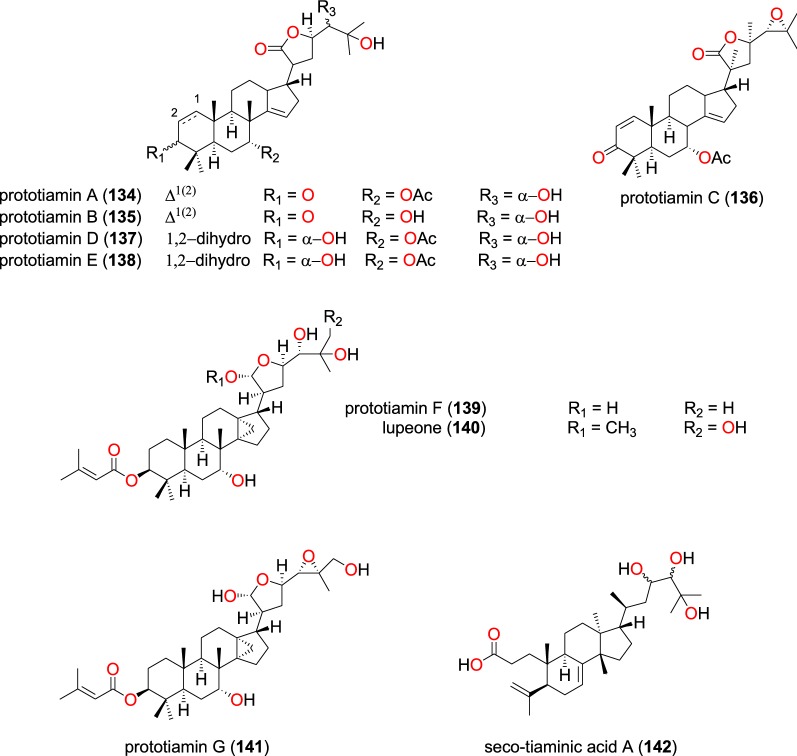
Apotirucallane triterpenoids (**134** to **142**)

Phytochemical investigations of the root barks of *Entandrophragma congoense* (Meliaceae) harvested from Kenya, led Happi et al. to the isolation of the novel apotirucallane triterpenoids with antiplasmodial activities; prototiamins A–F (**134**–**139**) (Fig. 29), as well as the known lupeone (**140**), prototiamin G (**141**) and seco-tiaminic acid A (**142**) [101]. The obtained compounds (**134**–**142**) were also evaluated against the CQ-sensitive strain NF54. Compound **134** displayed strong selectivity for the NF54 strain against rat skeletal myoblast L6 cells (with a selectivity index of 104.7), while **136** and **138** had selective indices of 12 and 13, respectively. Compounds **135**, **137**, **139**, and **140** were active against *P. falciparum*, with IC_50_ values ranging from 1.3 to 2.0 μM, and were less selective, while compound **142** inhibited the strain with an IC_50_ value of 19.3 μM.

##### Cycloartane triterpenes

**Fig. 30:**
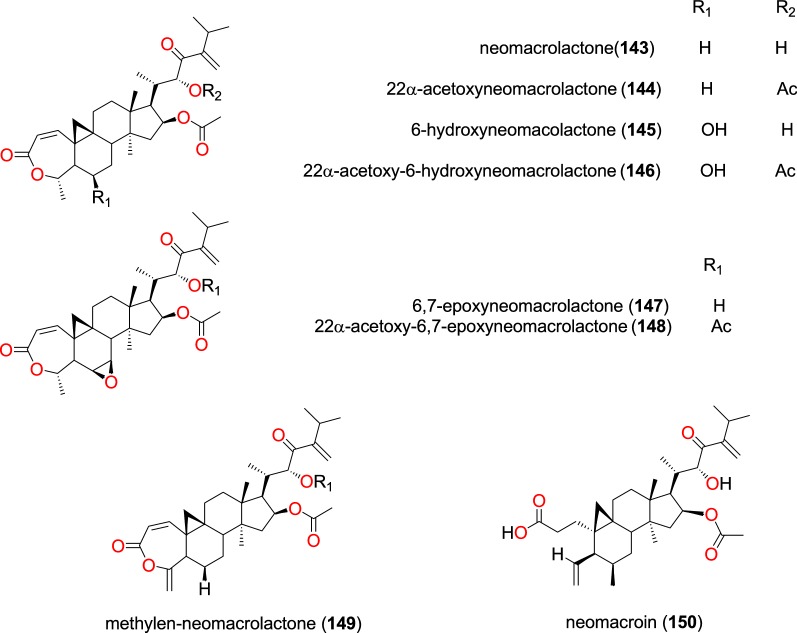
Cycloartane triterpenes (**143** to **150**)

The plant species *Neoboutonia macrocalyx* (Euphorbiaceae) is traditionally used to treat malaria in Southwestern Uganda around Kibale National Park, where the stem bark is widely used [96]. The investigation of the stem bark of this plant by Namukobe et al. led to the isolation nine new cycloartane triterpenes, among which eight; neomacrolactone (**143**), 22α-acetoxyneomacrolactone (**144**), 6-hydroxyneomacolactone (**145**), 22α-acetoxy-6-hydroxyneomacrolactone (**146**), 6,7-epoxyneomacrolactone (**147**), 22α-acetoxy-6,7-epoxyneomacrolactone (**148**), 4-methylen-neomacrolactone (**149**), and neomacroin (**150**), Fig. 30, displayed anti-malarial properties [96]. The obtained compounds were also evaluated for antiplasmodial activity against the FcB1/Colombia strain and for cytotoxicity against the KB (nasopharyngeal epidermoid carcinoma) and MRC-5 (human diploid embryonic lung) cells. Compounds (**143**–**147**, **149**,**150**) exhibited antiplasmodial activities with IC_50_ of < 11 μM [96].

##### Lanostane triterpene and limonoids

**Fig. 31:**
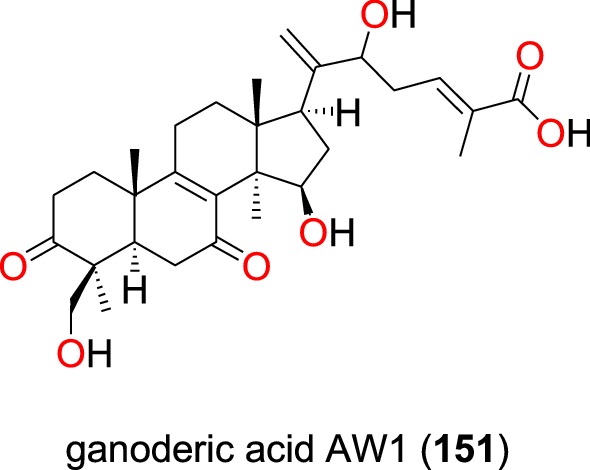
Lanostane triterpene (**151**)

Ganoderic acid AW1 (**151**) (Fig. 31), a new lanostane triterpene, was isolated from the whole organism of the *Ganoderma* sp. (Ganodermataceae) collected from Egypt [102]. This compound exhibited good anti-malarial activity against the D6 strain of *P. falciparum* with an IC_50_ value of 257.8 nM with no cytotoxicity up to the concentration of 9 μM. The compound also tested positive against the W2 strain with an IC_50_ value of 2000 nM [102].

**Fig. 32:**
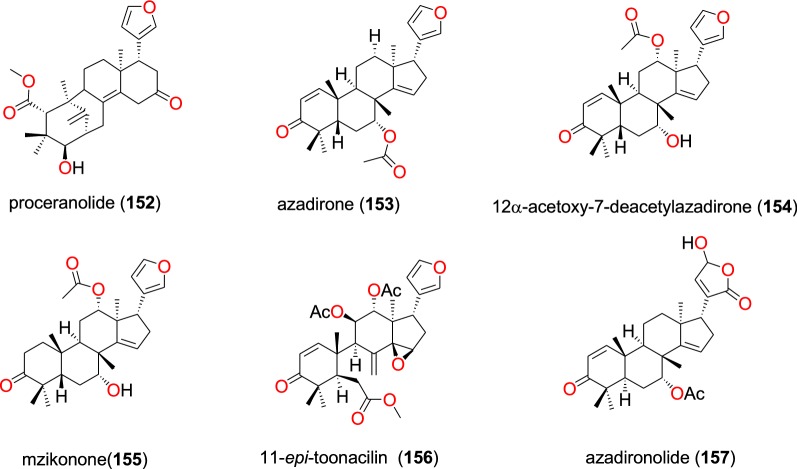
Limonoids (**152** to **157**)

The known limonoid, proceranolide (**152**) (Fig. 32) was found in the leaves of *Ekebergia capensis* (Meliaceae) by Irungu et al. [80]. The isolated compound was then evaluated in vitro against the D6 and W2 strains of *P. falciparum*. This compound exhibited weak antiplasmodial activity against the D6 and W2 strains with IC_50_ values of 84.7 and 150.2 µM, respectively [80].

Additionally, three known limonoids; azadirone (**153**), 12α-acetoxy-7-deacetylazadirone (**154**), mzikonone (**155**), 11-*epi*-toonacilin (**156**) and azadironolide (**157**), which were isolated from the stem bark of *Turraea nilotica* (Meliaceae), all showed potent antiplasmodial activity against the D6 and W2 strains with IC_50_ values ranged from 2.4 to 36.6 µM and from 1.1 to 40.5 µM, respectively [95].

##### Oleanane triterpenes

**Fig. 33:**
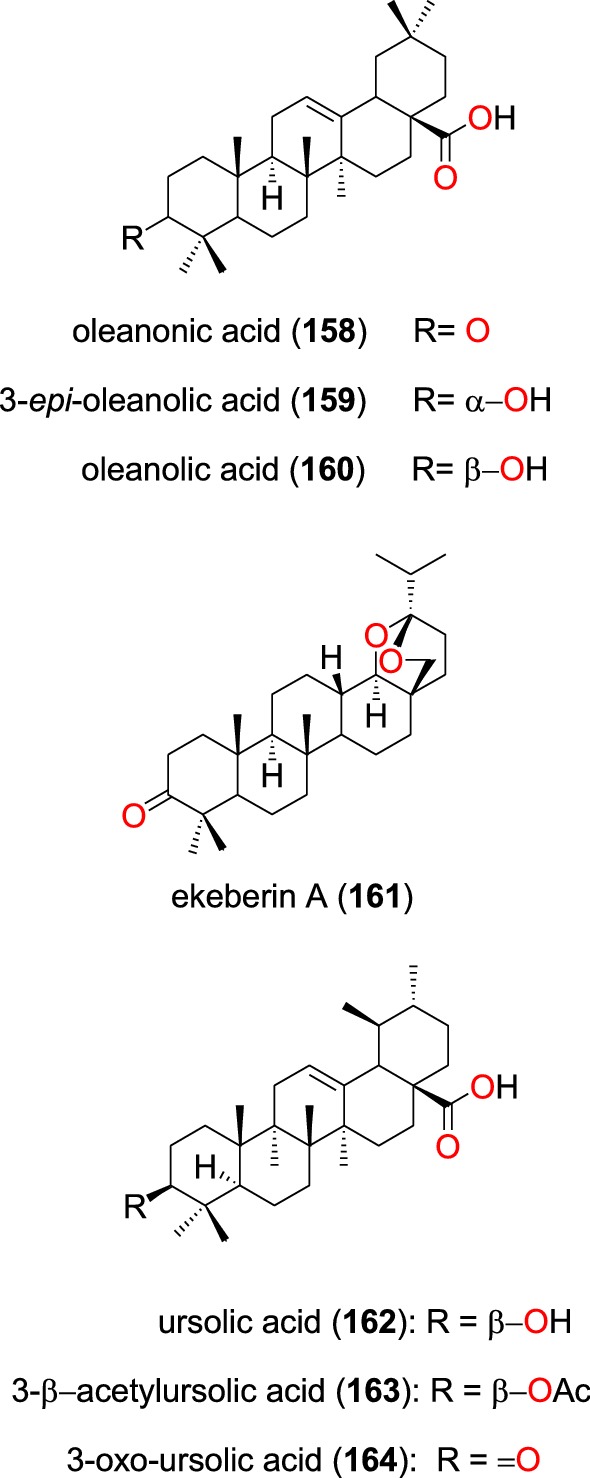
Oleanane triterpenes (**158** to **164**)

The known oleanonic acid (**158**), 3-*epi*-oleanolic acid (**159**), oleanolic acid (**160**) and ekeberin A (**161**) (Fig. 33) were also isolated from the leaves of *Ekebergia capensis* by Irungu et al. [80]. The four oleanane triterpenes potently inhibited the D6 and W2 strains of *P. falciparum* with IC_50_ values ranging from 38.8 to 205.0 µM and from 76.7 to 179.4 µM, respectively, against both strains.

Bero et al. also isolated the known ursolic acid (**162**) and oleanolic acid (**160**) from the twigs of *Keetia leucantha* (Rubiaceae). The authors re-tested the compounds, showing them to have in vitro activities on the 3D7 strain of *P. falciparum* with IC_50_ values of 32.4 and 59.4 µM, respectively [104]. From the leaves of *Mimusops caffra* (Sapotaceae) growing in South Africa, ursolic acid acetate (**163**) and 3-oxo-ursolic acid (**164**), as well as the known compound **162** were isolated by Simelane et al. [104]. These three compounds showed promising in vitro activities against the D10 strain with IC_50_ values ranging from 3.81 to 15.54 μM [104].

##### Tirucallane-type triterpenoids

**Fig. 34:**
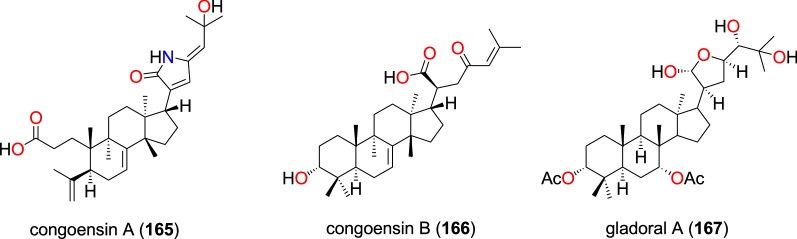
Tirucallane-type triterpenoids (**165** to **167**)

Two new tirucallane-type triterpenoids, namely congoensin A (**165**) and congoensin B (**166**), along with the known tirucallane-type triterpenoid gladoral A (**167**) (Fig. 34) were isolated from the bark of *Entandrophragma congoënse* (Meliaceae) harvested from Cameroon by Happi et al. [105]. These compounds exhibited activities against the NF54 strain with IC_50_ values ranging from 2.4 to 6.1 µM [105].

##### Protolimonoids

**Fig. 35:**
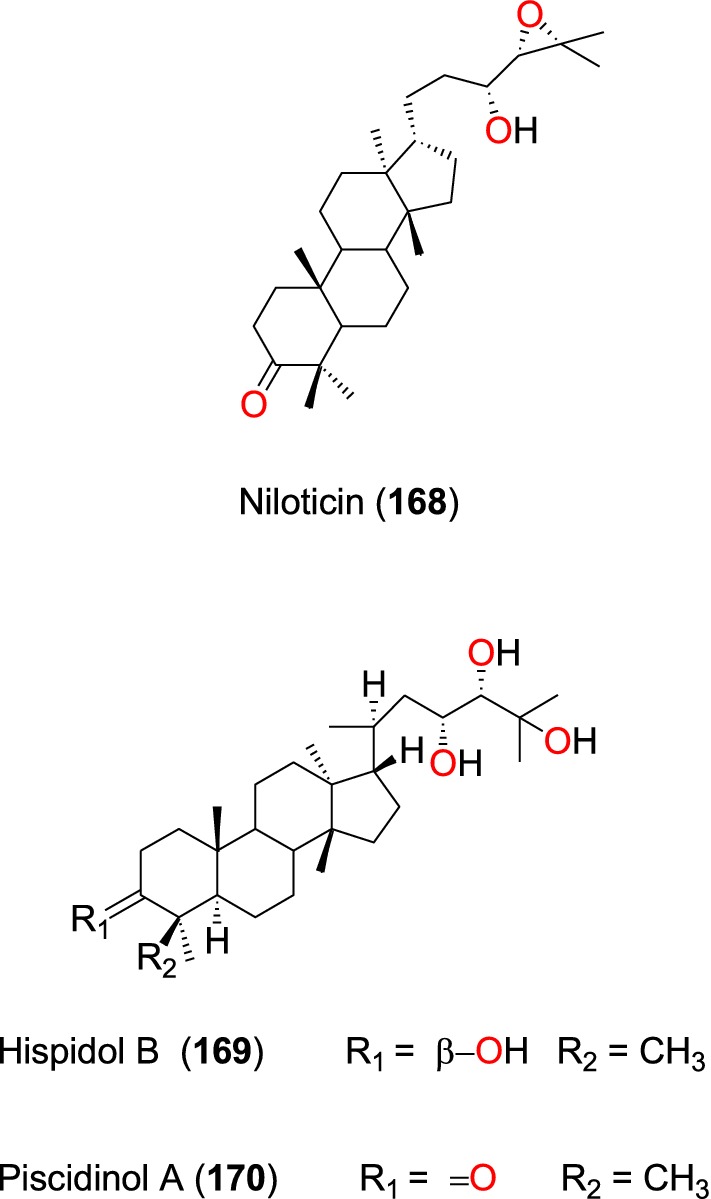
Protolimonoids (**168** to **170**)

Irungu et al. [95] also examined the stem bark of *Turraea nilotica* (Meliaceae) growing in Kenya. Three known potent anti-malarial protolimonoids; niloticin (**168**), hispidol B (**169**) and piscidinol A (**170**) were isolated (Fig. 35). These compounds exhibited activities against the D6 strain with IC_50_ values ranging from 36.8 to 48.2 µM and against the W2 strain, with IC_50_ values ranging from 37.2 to 77.0 µM [95].

##### Other triterpenoids

**Fig. 36:**
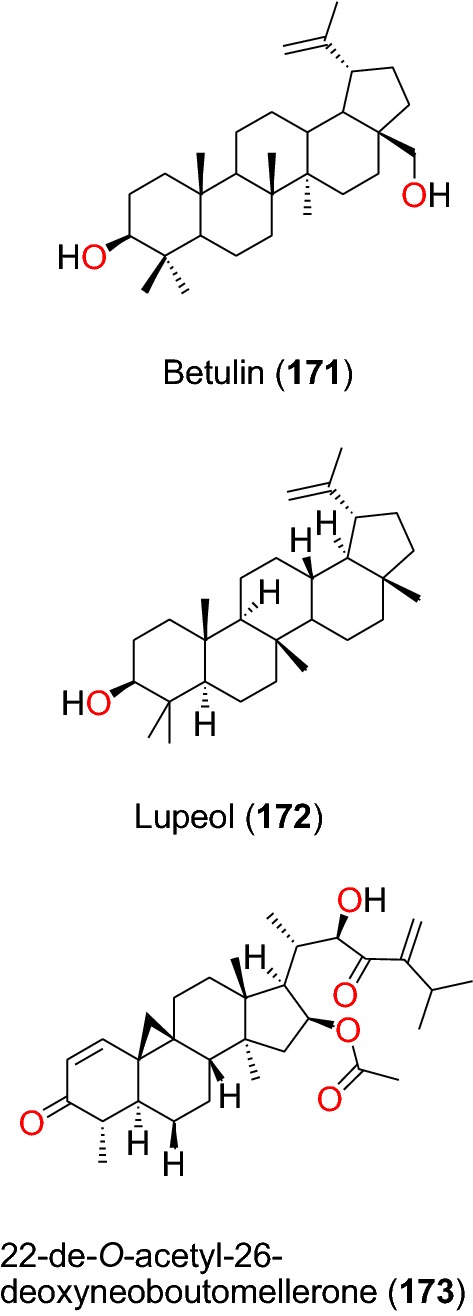
Other triterpenes (**171** and **173**)

The known hopane type triterpenoids; betulin (**171**) and lupeol (**172**) (Fig. 36) were isolated from the stem bark of *Diospyros canaliculata* (Ebenaceae) and *Erythrina caffra* (Papilionaceae), respectively [90, 106]. These triterpenoids only exhibited weak activities against the NF54 strain, with IC_50_ values of 112.94 and 97.73 μM, respectively [90, 106]. The cycloartane-type triterpenoid 22-de-*O*-acetyl-26-deoxyneoboutomellerone (**173**) was isolated from the stem bark of *Neoboutonia macrocalyx* (Euphorbaceae) [96]. The compound potently inhibited the CQ-resistant FcB1/Colombia strain of *P. falciparum*, with IC_50_ value of 2.15 µM [96].

### Other compound classes

**Fig. 37:**
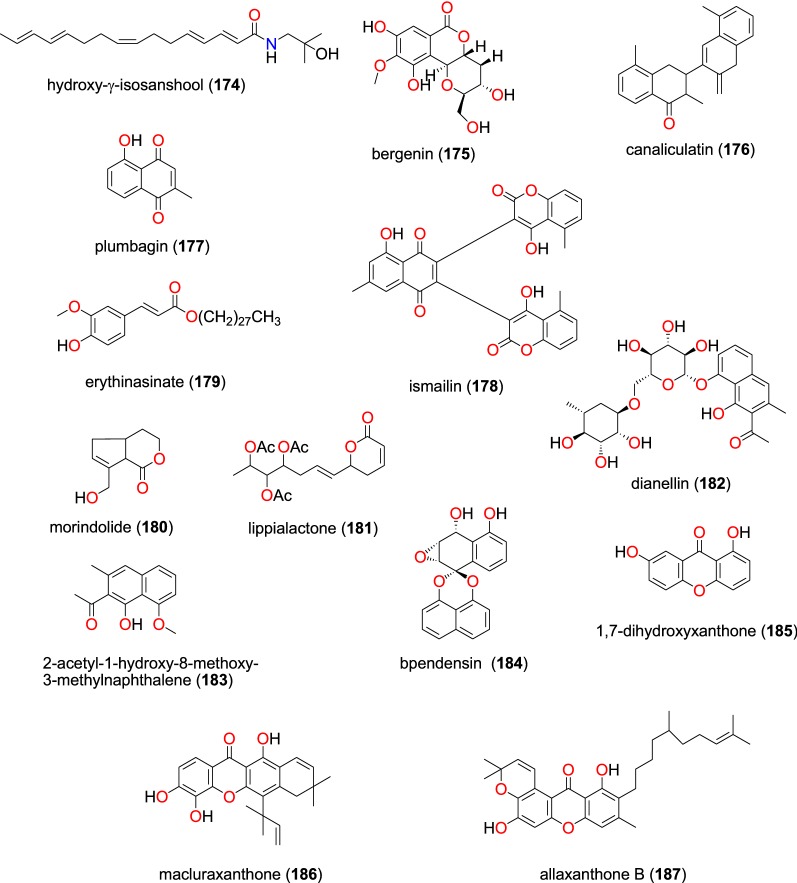
Other classes (**174** to **187**)

These are summarized in Table 8. The amide hydroxy-γ-isosanshool (**174**) and the coumarin bergenin (**175**), Fig. 37, obtained from the leaves of *Zanthoxylum heterophyllum* (Rutaceae) and *Diospyros conocarpa* (Ebenaceae), respectively [107, 108]. While the amide showed and activity against the 3D7 strain with IC_50_ = 39.04 µM [107], and percentage viability of compound **175** was recorded as 101.15 against the same plasmodial strain [108]. Lenta et al. also isolated three known coumarins; canaliculatin (**176**), plumbagin (**177**) and ismailin (**178**) from the stem bark of *Diospyros canaliculata* (Ebenaceae) harvested from Cameroon [90]. The compounds were shown to be active against the NF54 strain of *P. falciparum* with IC_50_ values ranging from 2.17 to 60.09 µM [90].

**Table 8:** Summary of other compound classes

Compound subclass	Isolated metabolites	Plasmodial strain (activities)	Plant species (Family), Taxon ID^a^	Part of the plant studied	Place of harvest (Locality, Country)	Author, references
Amide	**174**	3D7 (IC_50_ value = 39.04 µM)	*Zanthoxylum heterophyllum* (Rutaceae), NCBI:txid1908418	Leaves	Langevin, Reunion Island	Ledoux et al. [107]
Coumarins	**175**	3D7 (viability percentage = 101.15)	*Diospyros conocarpa* (Ebenaceae), NCBI:txid13492	Leaves, trunk, and roots	Ntouessong and Nkoemvone, Cameroon	Fouokeng et al. [108]
	**176**, **177** and **178**	NF54 (IC_50_ values vary from 2.17 to 60.09 µM)	*Diospyros canaliculata* (Ebenaceae), NCBI:txid13492	Stem bark	Kribi, Cameroon	Lenta et al. [90]
Ester	**179**	NF54 (IC_50_ value = 42.59 µM)	*Erythrina caffra* (Papilionaceae), NCBI:txid3842	Stem bark	Pietermaritzburg, South Africa	Chukwujekwu et al. [106]
Lactones	**180**	NF54 (IC_50_ value = 109.99 µM)	*Vangueria infausta* spp. *infausta* (Rubiaceae), NCBI:txid164485	Roots	Mutale Municipality, Limpopo Province, South Africa	Bapela [109]
	**181**	D10 (IC_50_ value = 24.70 µM)	*Lippia javanica* (Verbenaceae), NCBI:txid925357	Leaves	Thathe Vondo village, Limpopo Province, South Africa	Ludere et al. [110]
Naphthalene derivatives	**182** and **183**	D6 (IC_50_ value = 10.52 µM for compound **182**)	*Kniphofia foliosa* (Asphodelaceae), NCBI:txid214838	Rhizomes	Addis Ababa, Ethiopia	Induli et al. [85]
		W2 (IC_50_ value = 6.32 µM for compound **182**)				
		3D7 (IC_50_ value = 67.32 µM for compound **183**)				
	**182**	D6 (IC_50_ value = 10.48 µM)	*Kniphofia foliosa* (Asphodelaceae), NCBI:txid214838	Roots	Gedo, Ethiopia	Abdissa n [86]
		W2 (IC_50_ value = 6.28 µM)				
Spirobisnaphthalene	**184**	NF54 (IC_50_ value = 73.28 µM)	*Entandrophragma congoense* (Meliaceae), NCBI:txid2590899	Bark	Nkomokui, Cameroon	Happi et al. [101]
Xanthones	**185** to **187**	F32/24h (IC_50_ values range from 1.16 to 70.33 µM)	*Allanblackia floribunda* (Guttiferae- Clusiaceae), NCBI:txid469914	Whole plant	Mount Kala, Cameroon	Azebaze et al. [78]
		F32/72h (from 0.91 to 50.23 µM)				
		FCM29/24h (from 0.83 to 17.93 µM)				
		FCM29/24h (from 0.68 to 67.22 µM)				

^a^Identification number of the source species, derived from the NCBI Taxonomy database

The known ester erythinasinate (**179**) was isolated from the stem bark of *Erythrina caffra* (Papilionaceae) collected in South Africa by Chukwujekwu et al. [106] and inhibited the NF54 strain with an IC_50_ value of 42.59 µM. The antiplasmodial activities of two lactones: morindolide (**180**) and lippialactone (**181**), obtained from roots of *Vangueria infausta* spp. *infausta* (Rubiaceae) and the leaves of *Lippia javanica* (Verbenaceae), respectively, were also evaluated [109, 110]. Compound **180** only inhibited the NF54 strain weakly, with an IC_50_ value of 109.99 µM, while compound **181** inhibited the D10 strain moderately with an IC_50_ value of 24.70 µM [109].

Two naphthalene derivatives; dianellin (**182**) and 2-acetyl-1-hydroxy-8-methoxy-3-methylnaphthalene (**183**) isolated from the rhizomes of *Kniphofia foliosa* (Asphodelaceae) harvested in Ethiopia both inhibited the D6, W2, and 3D7 strains of *P. falciparum* with IC_50_ ranging from 6.32 to 67.32 µM [85, 86]. The spirobisnaphthalene bipendensin (**184**) was isolated from the bark of *Entandrophragma congoense* (Meliaceae) collected in Cameroon by Happi et al. [101], this naphthalene derivative inhibiting the NF54 strain with an IC_50_ value of 73.28 µM.

Three xanthones; 1,7-dihydroxyxanthone (**185**), macluraxanthone (**186**) and allaxanthone B (**187**) were obtained from *Allanblackia floribunda* (Guttiferae) by Azebaze et al. [78]. The three compounds exhibited antiplasmodial activities against the F_32_ and FCM_29_ strains with IC_50_ values ranging from 0.91 to 70.33 µM for the first strain and from 0.68 to 67.22 µM against the second [78].

### Novel compounds identified and principal compound classes

**Fig. 38:**
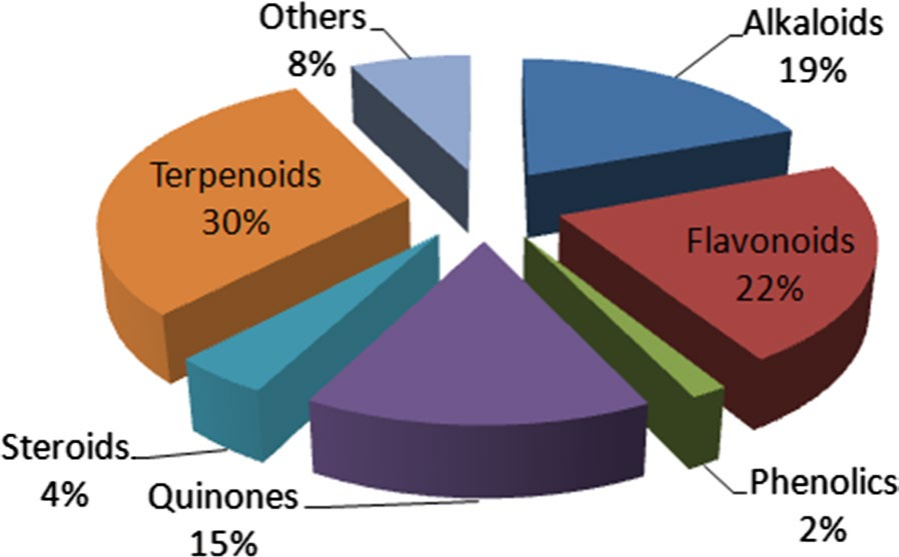
Pie chart showing the distribution of the 187 NPs by compound classes

**Fig. 39:**
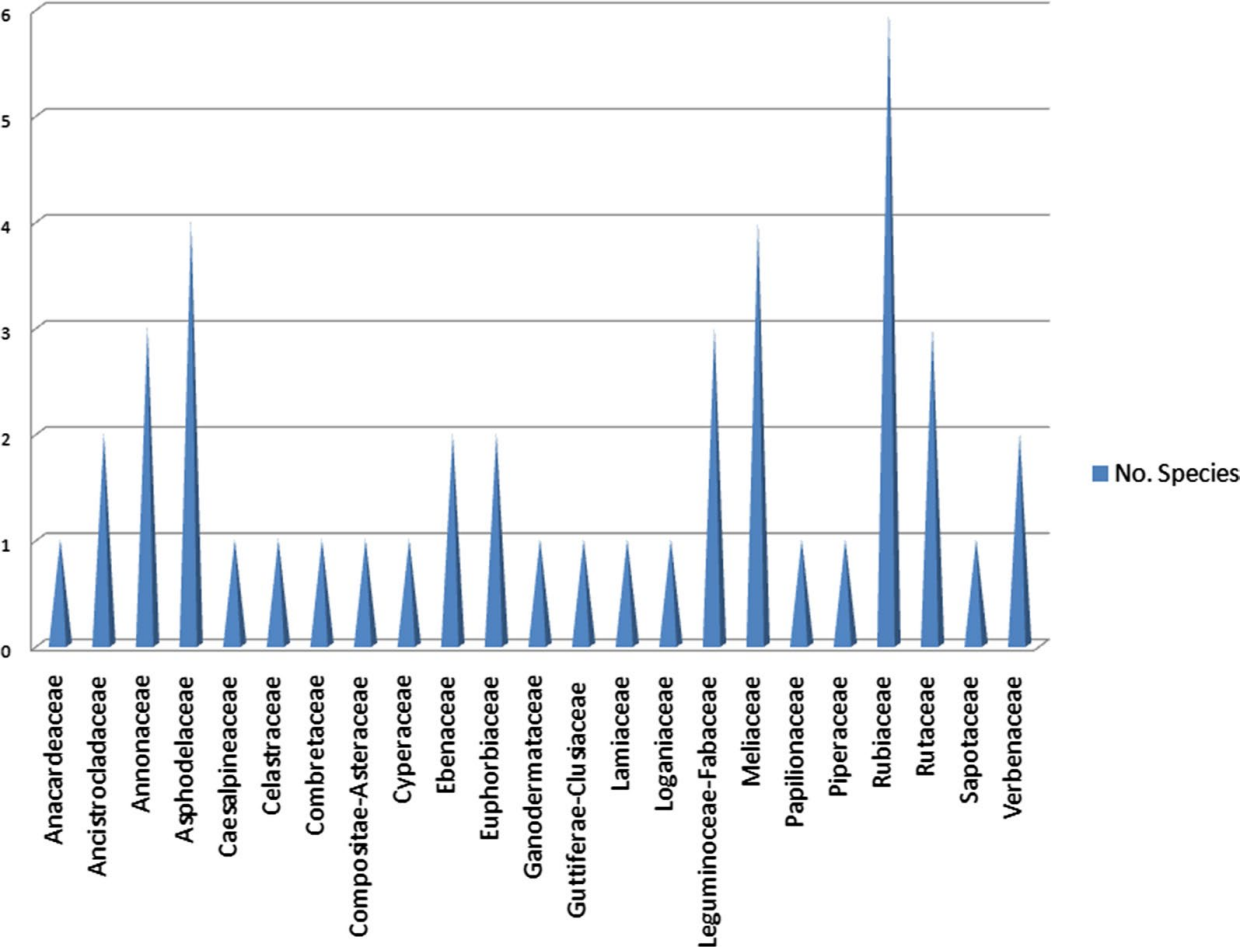
Bar chart showing the distribution of the number of plant species of origin by their families

It was observed that 53 out of the 187 compounds (about 28%) were described in the literature for the very first time. Besides, from Fig. 38, the majority of the NPs were terpenoids (30%), followed by flavonoids (22%), alkaloids (19%) and quinones (15%), the rest of the compound classes, each representing only less than 5% of the entire compound collection. It was also observed that most of the plant species from which the compounds were identified were of the families Rubiaceae, Meliaceae, and Asphodelaceae (Fig. 39).

### Compound distribution by plant families

**Fig. 40:**
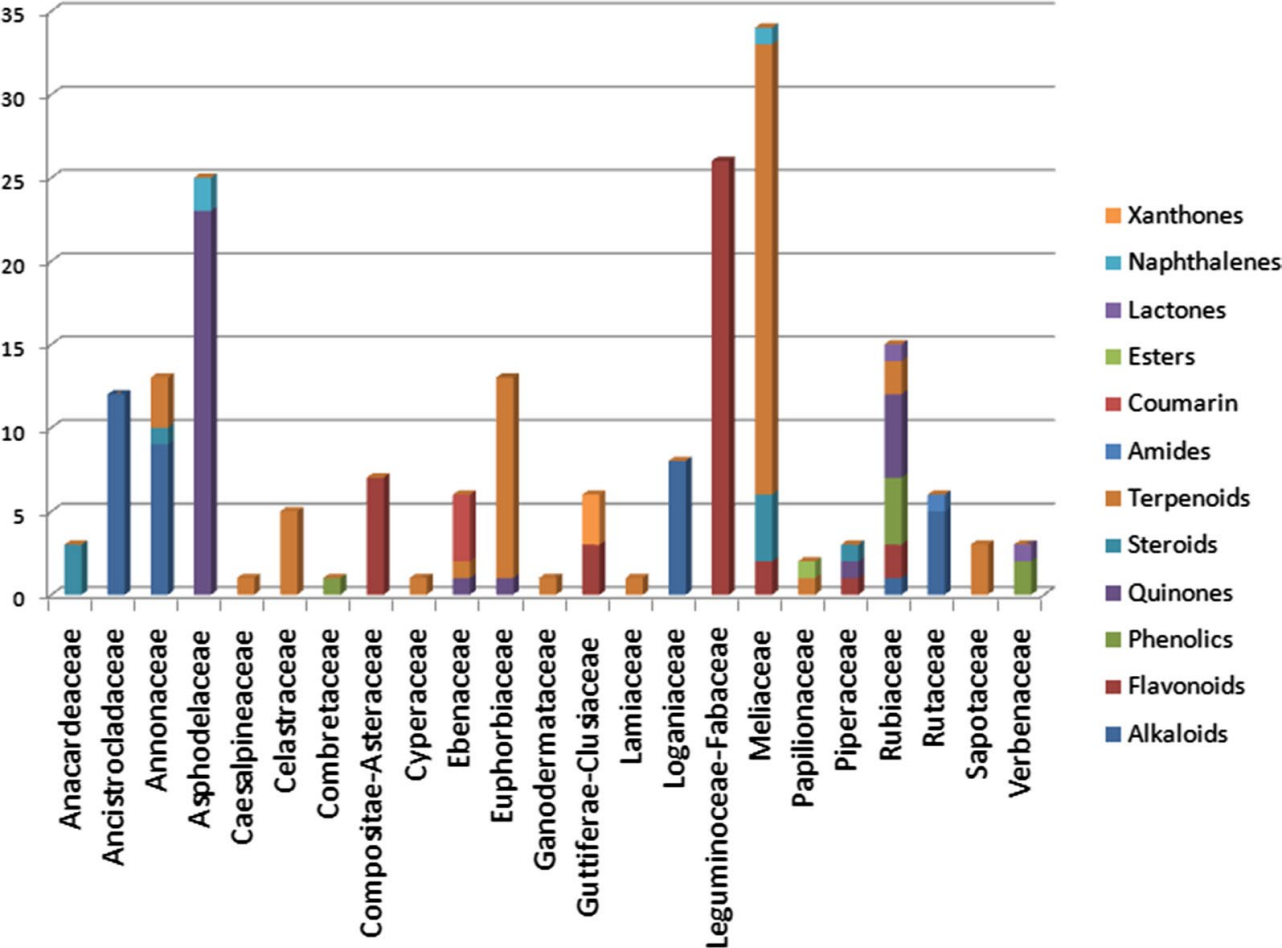
An attempted chemotaxomic distribution of compound by their classes sorted by the families of the plant species from which they were identified

A classification of the compounds by class into the plant families showed that most of the plant families represented their typical (chemotaxonomic) compound classes, often seen in the literature for species harvested from the African continent [34, 111–114]. As an example, for the collected data (Fig. 40), all the 26 compounds from the Leguminoceae-Fabaceae were flavonoids, while 23 out of the 25 anti-malarial NPs from the Asphodelaceae were quinones. It was also noted that 27 out of the 34 compounds from the Meliacious species were terpenoids, just like the Euphorbiaceous species that included 12 terpenoids out of 13 compounds identified within the family. Meanwhile, all the 12 compounds from the Ancistrocladaceae were alkaloids, just like the Loganiaceae and Annonaceae for which all 8 compounds and 9 out of the 12 identified compounds were, respectively, alkaloids. On the contrary, the compounds from the Rubiaceous species were distributed among different classes, the majority being phenolics and quinones.

### The most active compounds

Raw data retrieved from the literature showed activities reported in diverse units. A classification of the compounds by potencies (after all measured IC_50_ values were converted to μM), and taking a cut off of 10 μM for the most promising secondary metabolites most likely to be lead compounds. The most active compounds within this range for at least one plasmodial strain, i.e. 25 out of 66 NPs were alkaloids ~ (38%), while 23 of them were terpenoids ~ (35%) and 11 were quinones ~ (17%). Taking a cut off IC_50_ value of at most 1 μM left us with 19 compounds, 14 of them being alkaloids. Besides, the majority of the 187 NPs were terpenoids (30%), followed by flavonoids (22%), alkaloids (19%) and quinones (15%), the rest of the compound classes only represent a negligible part of the current collection.

## Conclusions

In this review, an attempt has been made to document the anti-malarial/antiplasmodial activities of NPs derived from African medicinal plants in their various compound classes and source species, published between 2013 and 2019. A description of the in vitro and available in vivo activities for 187 compounds is shown, as well as their classification into the various known NP compound classes and plant families of origin. From the collected data, the most active compounds belong to the same compound classes as the malarial drugs of natural origin, e.g. the alkaloid class for quinine and the terpenoid class for artemisinin. A previous report from Titanji et al. [115] had shown that plant-derived alkaloids from African medicinal plants have a great potential for anti-malarial drug development.

Although recently published reviews have described the activities of anti-malarial secondary metabolites of terrestrial and marine origins, input data from African sources has not been the focal point. Tajuddeen and van Heerden recently published a review of 1524 natural compounds from around the world, which have been assayed against at least one strain of *Plasmodium*, out of which 39% were described as new NPs, with 29% having IC_50_ values ≤ 3.0 µM against at least one of the tested plasmodial strains [116]. However, the study was limited to the period between 2010 and 2017 and did not include data from 2018 to 2019. Although the ability of NPs to block the transmission of malaria is still in the early stage, the current review, along with the previous studies that covers data for antiplasmodial compounds from African flora [27, 28], could serve as the baseline data for the discovery of new anti-malarial compounds from Africa.

## Supplementary information

**Additional file 1.** List of journals consulted in building the initial data collection.

## Data Availability

Not applicable.

## Abbreviations

ATM: African Traditional Medicine
AT: Artemisinin
CQ: Chloroquine
NP: Natural product
WHO: World Health Organization
