# Supplementary material for: The potential of anti-malarial compounds derived from African medicinal plants: a review of pharmacological evaluations from 2013 to 2019
Source: Malar J. 2020 May 18;19:183. doi: 10.1186/s12936-020-03231-7 (PMC7236213; doi:10.1186/s12936-020-03231-7)
Supplement: Supplementary file 1 — Additional file 1. List of journals consulted in building the initial data collection. [file 12936_2020_3231_MOESM1_ESM.doc]

# SUPPLEMENTARY MATERIAL

# The potential of anti-malarial compounds derived from African medicinal plants: a review of pharmacological evaluations from 2013 to 2019

Boris D. Bekono^1#^, Fidele Ntie-Kang^2,3,4,#,*^, Pascal Amoa Onguéné^5^, Lydia L Lifongo^1^, Wolfgang Sippl^3^, Karin Fester^6^, Luc C. O. Owono^1,*^

^1^Department of Physics, Ecole Normale Supérieure, University of Yaoundé I, P.O. Box 47, Yaoundé, Cameroon

^2^Department of Chemistry, Faculty of Science, University of Buea, P. O. Box 63, Buea, Cameroon

^3^Department of Pharmaceutical Chemistry, Martin-Luther University of Halle-Wittenberg, Kurt-Mothes Str. 3, 06120, Halle (Saale), Germany

^4^Institut für Botanik, Technische Universität Dresden, Zellescher Weg 20b, 01062 Dresden, Germany

^5^Department of Chemistry, University Institute of Wood Technology Mbalmayo, University of Yaoundé I, BP 50, Mbalmayo, Cameroon

^6^Faculty of Natural and Environmental Sciences, Zittau/Görlitz University of Applied Sciences, Theodor-Körner-Allee 16, 02763 Zittau, Germany

^#^These authors contributed equally and should be regarded as joint first authors

^*^ Corresponding authors

ntiekfidele@gmail.com (FNK); lcowono@yahoo.fr(LCOO)

List of Journals Consulted in Building the Initial Data Collection

| Journal type | List |
| --- | --- |
| International | *Acta Crystallographica*, *African Journal of Health Sciences*, *Asian Journal of Traditional Medicine*, *Biochemical Systematics and Ecology*, *Bioorganic Chemistry*, *Bioorganic and Medicinal Chemistry*, *Bioorganic and Medicinal Chemistry Letters*, *Bioscience Biotechnology and Biochemistry*, *BMC Complementary and Alternative Medicine*, *BMC Chemistry*, *BMC Research Notes*, *Boletín Latinoamericano y del Caribe de Plantas Medicinales y Aromáticas*, *Bulletin of the Chemical Society of Ethiopia*, *Carbohydrate Research*, *Chemical Data Collections*, *Chemical Papers*, *Chemistry and Biodiversity*, *Chemical and Pharmaceutical Bulletin*, *Fitoterapia*, *Helvetica Chimica Acta*, *Inflammopharmacology*, *Journal of Natural Products*, *Journal of Asian Natural Products Research*, *Journal of Ethnopharmacology*, *Journal of the American Oil Chemistry Society*, *Malaria Journal*, *Molecules*, *Natural Product Communications*, *Natural Product Letters*, *Natural Product Research*, *Natural Product Reports*, *Natural Product Science*, *Pakistani Journal of Medical Science*, *Parasitology Research*, *Pharmacologia*, *Pharmacologyonline*, *Pharmazie*, *Phytochemistry*, *Phytochemistry Letters*, *Pharmaceutical Biology*, *Phytotherapy Research*, *Phytomedicine*, *Planta Medica*, *PLoS One*, *Pure and Applied Chemistry*, *Rasayan Journal of Chemistry*, *Records of Natural Products*, *Research Journal in Phytochemistry*, *Scientific African*, *South African Journal of Botany*, *Tetrahedron*, *Tetrahedron Letters*  and *Zeitschrift für Naturforschung*. |
| Cameroonian | *Journal of the Cameroonian Academy of Sciences*, *Cameroon Journal of Biosciences* and *Les Annales des la Faculté des Sciences des l’Université de Yaoundé I.* |
